# Compression of Molybdenum Blue Polyoxometalate Cluster Rings

**DOI:** 10.1021/jacs.5c00187

**Published:** 2025-03-18

**Authors:** Vishal Lakhanpal, Melanie Guillén-Soler, Laia Vilà-Nadal, De-Liang Long, Leroy Cronin

**Affiliations:** School of Chemistry, University of Glasgow, University Avenue, Glasgow G12 8QQ, United Kingdom

## Abstract

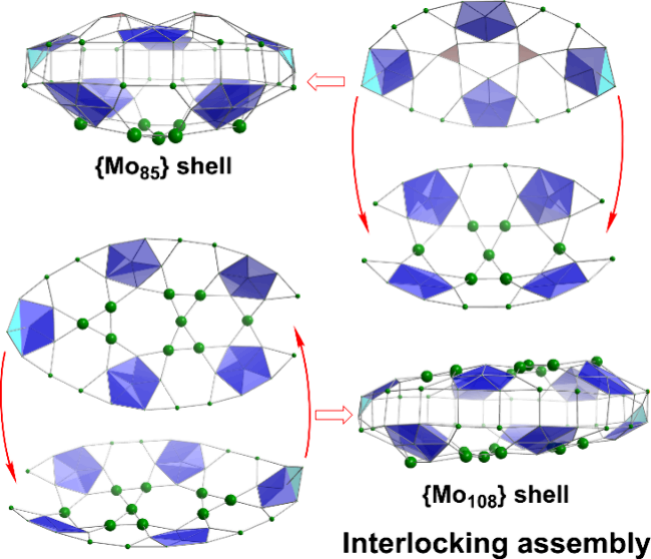

The self-assembly of polyoxometalate (POM) clusters remains challenging because they heavily depend on highly sensitive synthetic conditions that produce a vast library of potential building blocks and subunits such that explicit control is hard. This work reports new strategies to construct compressed molybdenum blue (MB) type cluster rings with a new range of giant MB POM clusters {Mo_54_}, {Mo_58_}, {Mo_85_}, and {Mo_108_}. These MB clusters prove the limits of the ring structure archetype, showing that it is possible to compress the ring by 100 metal atoms from 154 to 54 yet keep the electronic structure and ring shape. These structures comprise distorted pentagonal building blocks. The compression of the ring is achieved by using a {Mo_3_S} unit and {Mo_5_} bridging units. The {Mo_85_} and {Mo_108_} clusters exhibit a unique closed architecture, and redox studies demonstrate the reduced nature of these clusters.

## Introduction

Polyoxometalates (POMs) are a set of molecular metal oxides^1−6^ that are capable of displaying a diverse array of applications in materials^7−13^ and catalysis.^14,15^ One interesting subgroup of POMs are the giant “nanosized” polyoxomolybdate clusters, which can contain hundreds of molybdenum atoms and can be similar in size to proteins, bridging the gap between “traditional” molecular entities (<2 nm) and less precisely defined polymeric entities.^16,17^ Such giant clusters have unique properties^16,17^ and can also be further incorporated into even larger assemblies or high-dimensional materials.^18−23^ During the past three decades, there has been much growth in this area since specifically from 1996 when Müller et al. determined the first crystal structure of the giant molybdenum blue (MB) wheel {Mo_154_},^24^ a mixed-valent wheel-shaped Mo^V/VI^ oxide cluster comprising 154 Mo centers. Later, the scope of this was extended to the discovery of ball-shaped {Mo_102_}^25^ and {Mo_132_},^26^ wheel-shaped {Mo_176_},^27,28^ capped wheel {Mo_248_},^29^ and lemon-shaped {Mo_368_}.^30^ More recent advances include the discovery of the half-closed wheel {Mo_180_},^31^ neutral wheel-shaped {Mo_90_Ln_10_},^32^ the lantern-shaped L-{Mo_132_},^33^ capped wheel C-{Mo_132_},^34^ and their synthesis under electrochemical,^35^ flow,^36,37^ and high-temperature control.^32^

**Figure 1:**
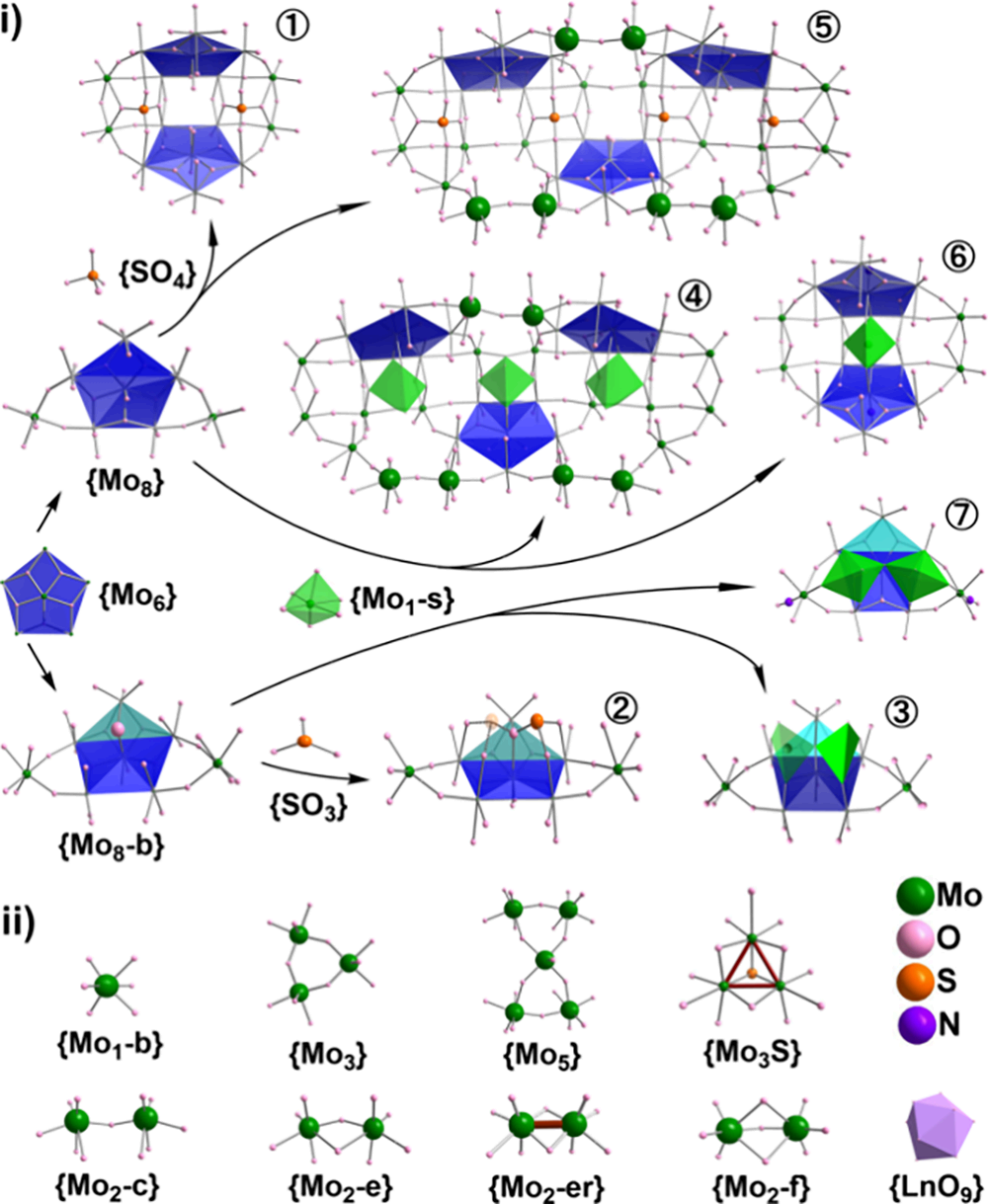
(i) Main building blocks: pentagon {Mo_6_}, normal pentagon based {Mo_8_} and bent pentagon based {Mo_8_-b}. Note that in {Mo_8_-b}, the pentagon central Mo shares an out-of-plane oxo ligand (enlarged sphere) with a peak Mo that causes the pentagon bending. Summary of MB construction schemes as supporting modes ①–⑦ of supporting units {SO_4_}, {SO_3_}, and {Mo_1_-s} to pentagon units. Modes ①–③ are new observations from this work. {SO_3_} and {Mo_1_-s} at the back of {Mo_8_-b} (modes ② and ③) each is divided into two equivalent positions, each with half occupancy; (ii) bridging units including reduced {Mo^IV^_3_S}, corner-sharing {Mo_2_-c}, edge-sharing {Mo_2_-e}, reduced edge-sharing {Mo^V^_2_-er}, face-sharing {Mo_2_-f}, and lanthanide ion {LnO_9_}. {Mo_5_} is newly observed in this work.

It is important to note that these aforementioned structures all share several common features: each structure contains transferable pentagonal {Mo_6_} or it is derivative {Mo_8_} groups as building blocks, together with variable supporting/templating and bridging units. Figure 1 summarizes the building units and construction schemes of various supporting/templating modes observed. {Mo_154_}^24^ and {Mo_176_}^27,28^ wheels are archetypal examples, consisting of 14 or 16 normal pentagon-based {Mo_8_} building blocks joined by equal numbers of supporting {Mo_1_-s} units at the backbone (most common supporting mode ④) and {Mo_2_-c} bridging units as skirt along the wheel edges.

The wheel sizes are controlled by the number of {Mo_2_-c} units. The fixed-sized {Mo_2_-c} units to complete the ring account for the limited accessibility of other structures of this type with smaller or larger ring sizes, other than {Mo_154_} and {Mo_176_}. Previously, it was shown that the smaller sized MBs are generated by replacing part or all of the {Mo_2_-c} units with smaller bridging units such as lanthanide (Ln) or uranyl ions as seen in the cases of {Mo_128_Ln_2_},^38^ {Mo_124_Ln_4_},^39−42^ {Mo_120_Ln_6_},^43,44^ {Mo_100_Ln_6_},^45^ {Mo_90_Ln_10_},^32,34^ and {Mo_90_U_10_},^46^ consisting of 12 or 10 {Mo_8_} building blocks. The Mo Brown {Mo_102_}^25^ and {Mo_132_}^26^ balls adopt a slightly different architecture with their frameworks built by 30 {Mo_1_-b} or {Mo_2_-er} bridging units to connect the 12 {Mo_6_} pentagons into the overall ball-shaped architectures. The formation of the MB clusters is a highly intricate assembly process.^47−50^ To efficiently engineer new MB species, an understanding of the building blocks generated under a given set of synthetic conditions is required, as well as the spatial arrangements, which they may occupy.^51^ Therefore, to explore a chemical space to engineer new MBs, it is essential to determine the conditions under which pentagon-based main building blocks {Mo_8_} and other accessary units are formed. Typically, the pentagonal {Mo_6_} units in the MB are least reduced and account for the majority of Mo^VI^. The minority of valence-variable Mo^V^ centers are located at backbones and bridging units with the delocalization of the remaining Mo^IV^ centers, which produces the highly intense blue colors.^30,52^ Normally, the pentagonal {Mo_6_} group adopts a planar arrangement,^53^ where the largest deviation of any peak of the pentagon from the plane formed by the five peaks is less than 0.4 Å and a given Mo center is bound to either 6 or 7 oxo or water ligands, bridging adjacent Mo centers. Presently, there is only one example of the unusual distorted pentagonal unit {Mo_8_-b} (bent pentagon, Figure1) observed in {Mo_51_V_9_},^54^ which features one Mo peak displaying a deviation of 0.8 Å from the main pentagonal plane caused by this peak sharing an out-plane oxo ligand with a Mo atom from the center of the pentagon, see Figure 1.

By incorporating other hetero anions such as sulfate, to act as supporters/templates, with supporting mode ⑤, see Figure 1, during the self-assembly process, a similar conventional wheel structure can be created, e.g., the assembly process of the {Mo_146_}.^55^ Given the lack of traditional MB species featuring a hetero supporting unit replacing the {Mo_1_-b} bound to the central Mo atom of a pentagonal unit, we have long hypothesized that this feature could be of exploitation to continue to grow this family by introducing hetero species such as other transition metal ions, i.e., Ti^4+^ and Zr^4+^. Thus far, attempts at substituting hetero metallic species into analogous positions have been unsuccessful.

However, with both SO_4_^2–^ and SO_3_^2–^ occupying these backbone positions in different clusters, grounds for further exploration to derive new members of this type of large asymmetric MB have been provided. Herein, we report a range of fundamentally new MB structures whereby the ring has been compressed, containing a range of 6 to 10 pentagons: {Mo_54_}, {Mo_58_}, {Mo_85_}, and {Mo_108_}. Further, we demonstrate that the SO_3_^2–^ can act as a supporting unit for the first time and has new coordination modes for SO_4_^2–^ and dividing {Mo_1_-s} in MB construction. For the {Mo_85_} and {Mo_108_} clusters, we show that the distorted pentagonal units of the {Mo_8_-b} unit play a key role in interlocking the rings in the self-assembly of new MB architectures. This also shows an unprecedented {Mo_5_} bridging unit, closing unconventional MB structures by spanning a wide void, giving clusters with unique MB backbones. The {Mo_108_} cluster is a relatively large iso-polyoxomolybdate, which has formed without the involvement of any hetero metal ions or hetero anions, a rarely observed example of MB structures after {Mo_154_}^24^ and {Mo_176_}.^27,28^ In this article, we explore the limits of the wheel formation presenting both highly compressed wheel structures and a new library of related clusters that should help the further exploitation of these structure types.

## Results and Discussion

Four new closely related MB clusters have been produced, giving rise to a new family of MBs with closed shells. The four new clusters:
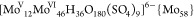
1
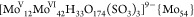
2
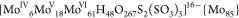
3and
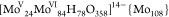
4were engineered via careful control of parameters such as pH, temperature, and amount of reducing agents to control the degree of reduction in the clusters. {Mo_58_} **1** was produced under hydrothermal conditions using heavily acidic conditions, where the final pH was 0.15. {Mo_54_} **2** was synthesized utilizing the same reagents, and concentrations, as **1**; however, the final pH was 2.8 and the reaction was not heated. {Mo_85_} **3** was formed under hydrothermal synthesis similar to **1** with less acidic conditions, where the final pH was 2.5. {Mo_108_} **4** was obtained under hydrothermal conditions, where the synthetic conditions differed from those of **1** – **3**. **4** was synthesized at pH 1.05 and was heated at 120 °C for 72 h.

**Figure 2:**
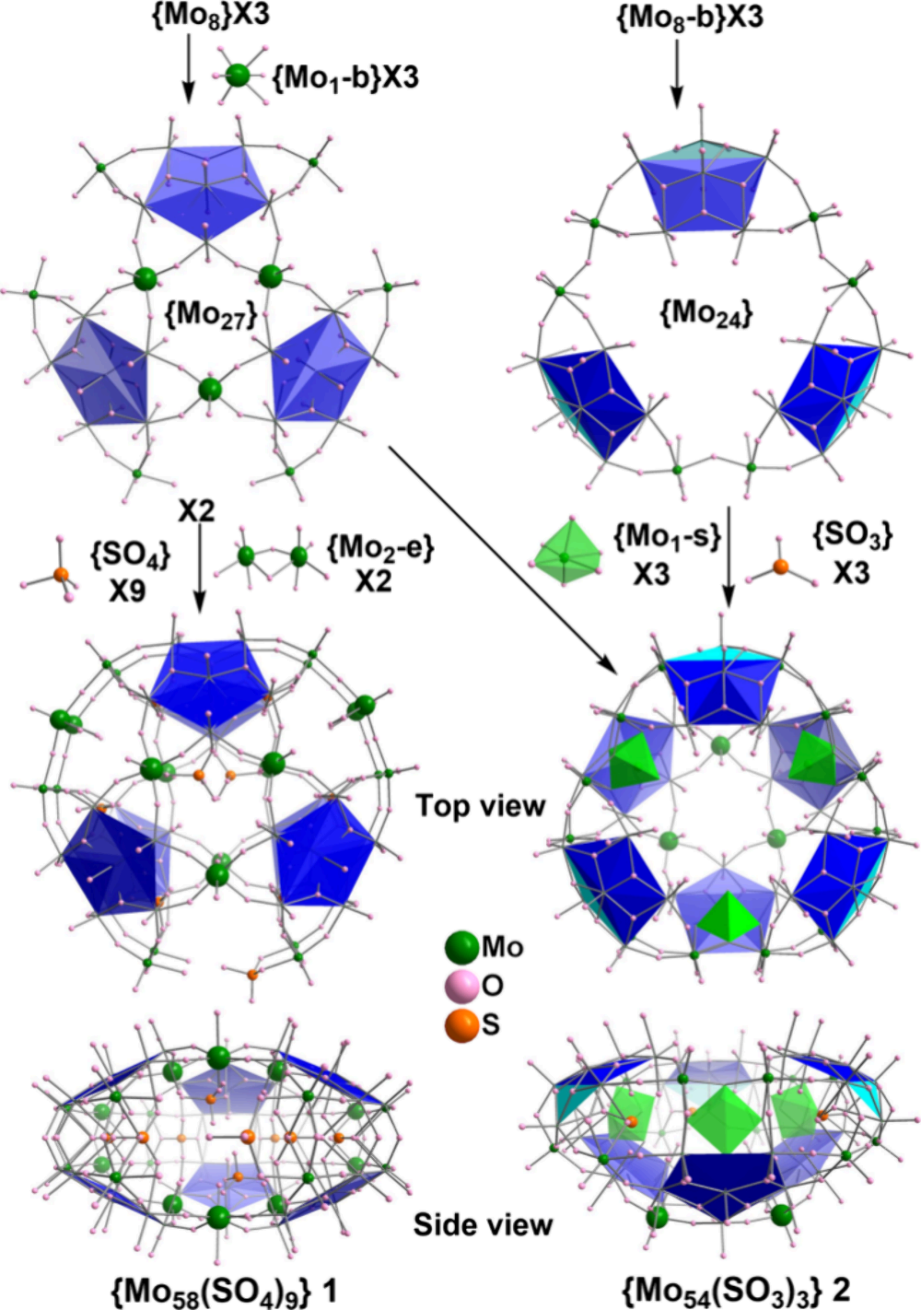
Schematic representation of the construction of {Mo_58_} **1** and {Mo_54_} **2** from the normal pentagon based {Mo_8_} and bent pentagon based {Mo_8_-b} building blocks with aids of the supporting units SO_4_^2–^ in mode ① and SO_3_^2–^ in mode ②.

**Figure 3:**
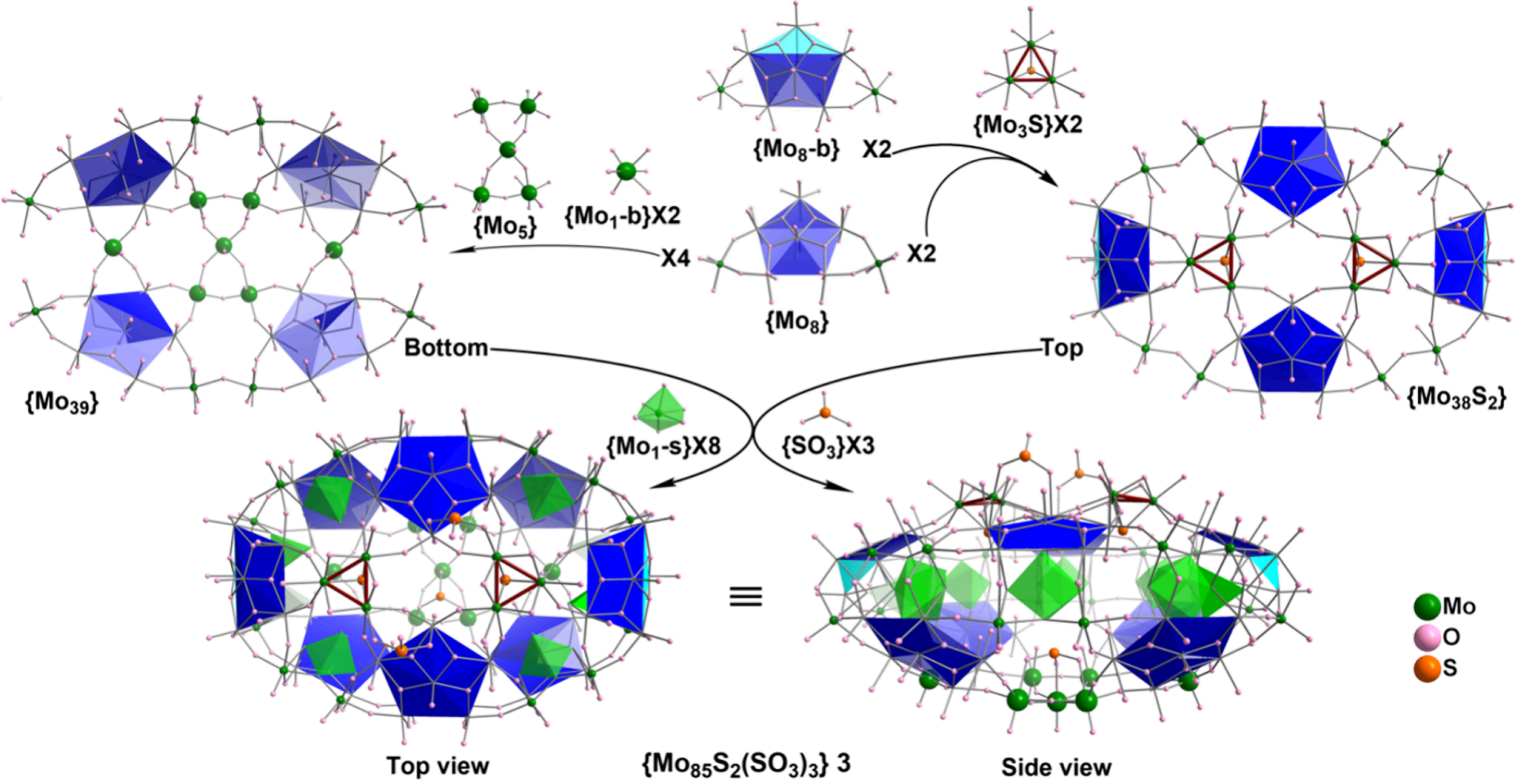
Schematic representation of the construction of {Mo_85_} **3** from the normal pentagon based {Mo_8_} and bent pentagon based {Mo_8_-b} building blocks with aids of the bridging units {Mo_1_-b}, {Mo_5_}, and {Mo_3_S}. Top {Mo_38_S_2_} and bottom {Mo_39_} are joined by eight {Mo_1_-s} supporting units within the equatorial backbone.

**Figure 4:**
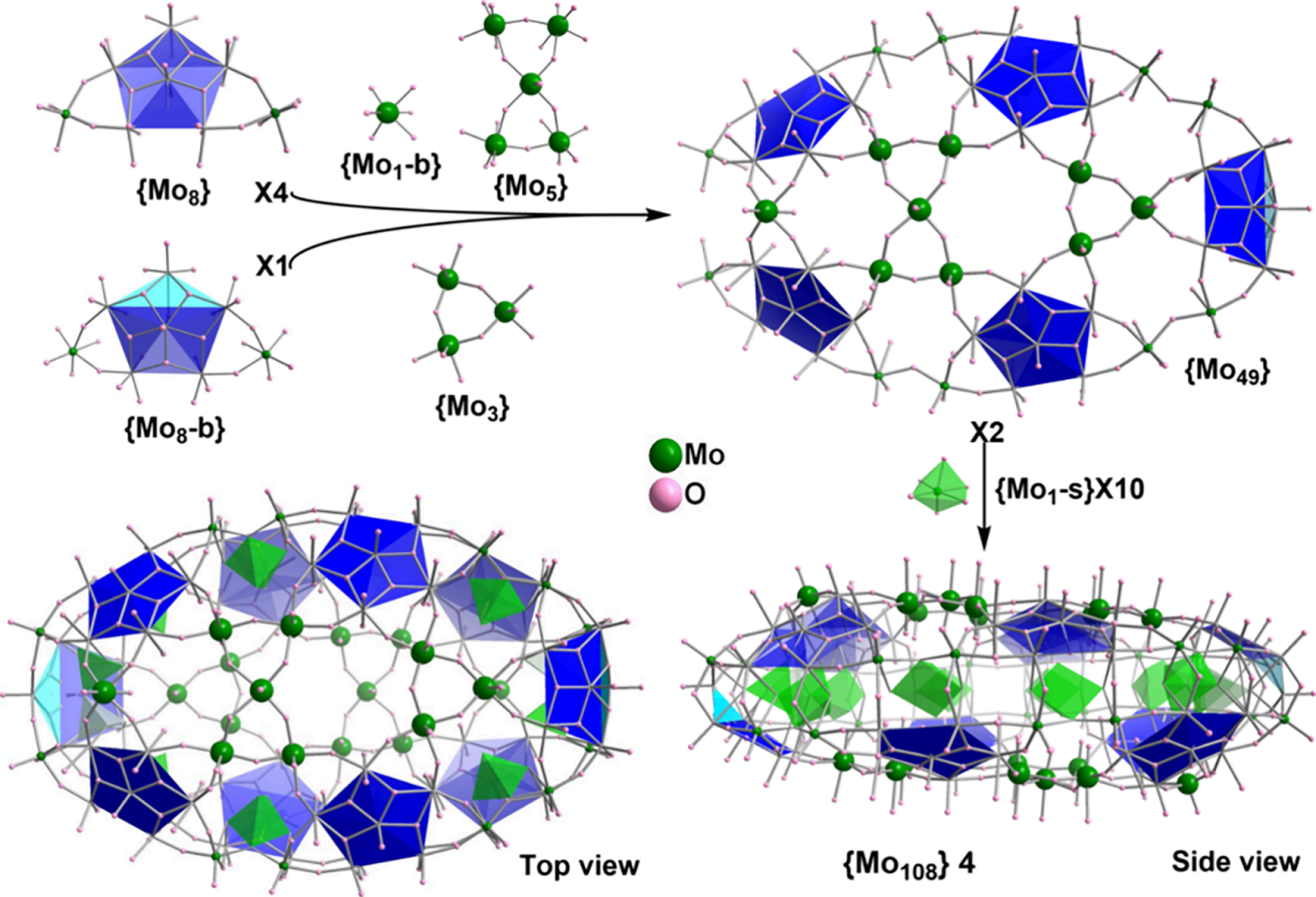
Schematic representation of the construction of {Mo_108_} **4** from the normal pentagon based {Mo_8_} and bent pentagon based {Mo_8_-b} building blocks with aids of the bridging units {Mo_1_-b}, {Mo_5_}, and {Mo_3_}. Two {Mo_49_} parts in a head-to-tail array are joined together by 10 {Mo_1_-s} supporting units inside the ellipsoid equatorial backbone. The bent peak Mo of {Mo_8_-b} reaches the middle of the equator and perfectly seal each other’s notch at the other end, forming a flattened ellipsoidal {Mo_108_}.

The formulas of **1**–**4** have been established by a series of analytical techniques including elemental analysis, redox titrations, thermogravimetric analysis (TGA), and bond valence sum analysis (BVS) in addition to single-crystal X-ray diffraction (SCXRD) analysis. **1**–**4** range in nuclearity and more interestingly in the number of pentagons, which produce their main framework: **1** and **2** possess the same number of pentagons, 6, but with a significantly different arrangement, overlapping or staggered (Figure 2), **3** contains 8 pentagons (Figure 3), and **4** has 10 pentagons (Figure 4). Despite this range in the number of pentagons, it is apparent that the clusters share architectural motifs, with **1**–**4** possessing unprecedented backbone construction schemes. In addition to this, **3** and **4** contain a {Mo_5_} bridge virtual building block, composed of two corner sharing {Mo_2_-c} and a {Mo_1_-b} bridging unit, which to our knowledge has not been observed as a building unit in the self-assembly of giant POMs before. This unit acts as a part of the capping group toward the closed shell assembly of **3** and **4**.

SCXRD revealed that {Mo_58_} **1** crystallizes as Na_2_H_4_·**1**·(H_2_O)_35_ in the orthorhombic system with centrosymmetric space group *Cmcm*. **1** adopts a trefoiled architecture comprising a C_3v_ symmetric {Mo_27_} hemisphere consisting of three normal {Mo_8_} building blocks and three {Mo_1_-b} bridging units, Figure 2. {Mo_58_} **1** doubles this {Mo_27_} hemisphere to become an oblate spheroid possessing horizontal mirror symmetry. This results in the formation of an equatorial symmetric backbone supported by six sulfate {SO_4_} units inside the spheroid in supporting mode ①. These units are of cis fashion, where two pentagons are on the same side of the {SO_4_} unit and overlap, as shown in Figure 1. Sulfate {SO_4_} as supporting units have been observed in the {Mo_146_} wheel structure^55^ in trans supporting mode ⑤, Figure 1, where pentagonal units are located in a staggered fashion, alternatively in up and down half rings. {Mo_58_} **1** is partially completed with two edge-sharing {Mo_2_-e} units (Figure 1) filling in two of the three notches, leaving the third notch unfilled and open. The Mo···Mo separation of 8.0 Å between two {Mo_8_} units at the unfilled notch is significantly larger than those of 7.5 Å at the filled notches (Figure S5A). The 0.5 Å difference implies that the gap is too big to be bridged up by a third {Mo_2_-e} unit, and it is unlikely to obtain a complete oblate spheroid for the current backbone assembly.

Similar trefoiled architectures have previously been reported involving smaller hetero metal ions to replace the {Mo_1_-b} bridging units or some Mo atom in {Mo_8_} building blocks, including {Mo_57_V_6_}^56^ and {Mo_57_Fe_6_},^57^ where three face sharing {Mo_2_-f} units (Figure 1) are located inside clusters between pentagon centered arcs to stabilize the structures. Meanwhile, the {Mo_1_-s} units in {Mo_57_V_6_}^56^ and {Mo_57_Fe_6_},^57^ are uniquely in mode ⑥, with each supporting two overlapped pentagons up and down simultaneously (Figure 1). At the defect notch in {Mo_58_} **1**, a sulfate anion was identified to coordinate to Mo centers on one side. Also, two other sulfate ligands were found inside the cluster bridging a pentagon peak and {Mo_1_-b} to reinforce the uncompleted defect oblate spheroid. BVS revealed that the cluster is 12 electrons reduced, where the delocalization of this charge, with 20% of Mo centers reduced to Mo^V^, giving rise to the typical blue color associated with MBs.

{Mo_54_} **2** crystallizes as Na_4_(NH_4_)_5_·**2**·(H_2_O)_50_ in the orthorhombic system with centrosymmetric space group *Pnma*. {Mo_54_} **2** shares a hemispherical construction with {Mo_58_} **1** and adopts a tapered hemispherical architecture, possessing two flat faces, Figure 2. In addition to the {Mo_27_} hemisphere mentioned in **1** as the bottom unit (Figure 2), a {Mo_24_} section was found as the top unit to form a tapered spherical bowl-like structure, Figure 2. The {Mo_24_} hemisphere consists of three bent pentagon-based {Mo_8_-b} units without {Mo_1_-b} bridges to close but show a much bigger window than that of {Mo_27_}. Below each {Mo_8_-b} unit is a sulfite {SO_3_} supporting unit, which is divided into two equivalent positions in mode ② shown in Figure 1. This is the first known example of a sulfite group to template an MB, although this has previously been reported in small POM cluster {Mo_18_(SO_3_)_2_}^58^ and large Mo red {Mo_240_}.^59^ As depicted in Figure 2, inside the {Mo_54_} backbone, there are three other {Mo_1_-s} supporting units each being behind one of the {Mo_8_} building blocks of the {Mo_27_} hemisphere in mode ④, which is the most popular mode as observed in {Mo_154_}^24^ and {Mo_176_}.^27,28^ Accordingly {Mo_54_} **2** has both {SO_3_} and {Mo_1_-s} supporting units in one structure alternatively arranged inside its backbone, and this is the first observation of this type, to the best of our knowledge. As such, the whole cluster of {Mo_54_} **2** has C_3v_ symmetry disregarding the disorders of sulfite anions. From the side view of {Mo_54_} in Figure 2, it can be seen that the bent peaks of the {Mo_8_-b} units reach the middle of the cluster equator and immaculately bridge the gaps on the bottom trefoiled {Mo_27_} hemisphere, forming a closed backbone, which contrasts to {Mo_58_} **1**. The cluster contains an internal cavity of approximately 8.6 Å in diameter with two windows: a 12-membered {Mo_6_O_6_} ring at the bottom and a 24-membered {Mo_12_O_12_} ring at the top. BVS indicates that the cluster is 12 electrons reduced, indicating that 22% of Mo centers are Mo^V^.

{Mo_85_} **3** crystallizes as Na_4_H_12_·**3**·(H_2_O)_85_ in the orthorhombic system with space group *Cmcm*. Figure 3 shows the ellipsoidal shell architecture of {Mo_85_} **3** constructed by two nonequivalent half shells, with top {Mo_38_S_2_} and bottom {Mo_39_}, joined by 8 {Mo_1_-s} supporting units, each located behind a pentagonal unit inside the equatorial backbone. Three sulfite ligands were found either inside the cluster or on the outside surface bridging pentagons and {Mo_5_} units to reinforce the flatted prolate spheroid. The top {Mo_38_S_2_} unit is composed of two normal {Mo_8_} and two bent {Mo_8_-b} building blocks alternatively arranged in an ellipse. Two {Mo_3_S} triads function as bridges linking up pentagons and closing the two big windows. The Mo centers in {Mo_3_S} triads are found in the +4 oxidation state, each involving two Mo–Mo single bonds of about 2.7 Å. Similar {Mo_3_S} triads in MB constructions were reported in the lantern structure L-{Mo_132_}.^33^ The bottom {Mo_39_} purely consists of four normal {Mo_8_} building blocks bridged up by two {Mo_1_-b} and one {Mo_5_} units. The observation of an {Mo_5_} bridging unit spanning the entire wide gap to close an MB cage is unprecedented in POM chemistry. The bent peak Mo atom of the {Mo_8_-b} in the top {Mo_38_S_2_} shell reaches the equator and perfectly fills the gaps left at the two ends of the bottom {Mo_39_} piece (Figure S9). Behind each {Mo_8_-b} pentagon, there is a {Mo_1_-s} supporting unit with the Mo atom divided over two equivalent positions each with half occupancy, supporting mode ③, in Figure 1. A similar arrangement was seen in {Mo_51_V_9_}^54^ but two Mo sites were reported with full occupancy, supporting mode ⑦, Figure 1. The narrower space inside the apex of the {Mo_85_} **3** ellipsoidal architecture may give rise to single occupancy instead of accommodating two Mo atoms simultaneously. BVS calculation indicates that the {Mo_85_} is composed of six Mo^IV^, 18 Mo^V^, and 61 Mo^VI^, indicating that the cluster is approximately 23% reduced excluding 6 Mo^IV^ on the two {Mo_3_S} bridging units.

{Mo_108_} **4** crystallizes as (NH_4_)_14_·**4**·(H_2_O)_95_ in the monoclinic system with centrosymmetric space group *C*2/*m*, displaying a remarkable closed shell structure. As shown in Figure 4, it is composed of 10 pentagon building blocks like {Mo_90_Ln_10_},^32^ but it does not form a wheel like MB and instead, the self-assembly process directs the cluster toward a closed ellipsoid, akin to {Mo_85_} **3**. Unlike {Mo_85_} **3**, which has two nonequivalent half sections, {Mo_108_} **4** has two centro-symmetrically related and identical top and bottom parts, {Mo_49_}, which are composed of four normal {Mo_8_} and one bent {Mo_8_-b} building blocks. A {Mo_1_-b}, a {Mo_3_}, and a {Mo_5_} unit located at their dedicated positions bridge up these pentagonal units and close the surface, forming a {Mo_49_} unit of half-vase shape, featuring different head and tail ends, Figure 4. Two {Mo_49_} parts in a head-to-tail orientation are joined together by 10 {Mo_1_-s} supporting units inside the ellipsoid equator. The bent peak Mo of the {Mo_8_-b} reaches the middle of the equator and interlocks the {Mo_49_} units at a notch position at opposing ends (Figure S9). Similar to those in {Mo_85_} **3**, the {Mo_1_-s} supporting units behind the bent {Mo_8_-b} are also each divided over two positions each with half occupancy, supporting mode ③. The {Mo_3_} triad present in {Mo_108_} **4** differs from {Mo_3_S} in {Mo_85_} **3**; the Mo centers are not fully reduced, and thus the individual Mo atom does not exhibit Mo–Mo bonding, and the triad is not capped by a hetero S^2–^ species. BVS calculations, verified by redox titration, show that **4** is 24-electrons reduced. The cluster possesses a large solvent-accessible internal cavity, with a vertex-to-vertex distance of about 21 Å and a covertex to covertex distance of about 14 Å. This cavity is accessible via a number of pores on either face of **4**, with the smallest accessible pore consisting of a window of a 12-membered {Mo_6_O_6_} cycle, and the largest possessing a 14-membered {Mo_7_O_7_} cycle.

A novel aspect of this work, common to all structures with the bent {Mo_8_-b} building blocks presented here in **2**–**4**, is the interlocking assembly that the bent peak of {Mo_8_-b} fills the notch formed between two normal {Mo_8_} building blocks (Figure S9). The ratios of {Mo_8_-b} to {Mo_8_} are 3:3, 2:6, and 2:8 in **2**, **3**, and **4**, respectively. Although normal {Mo_8_} building blocks solely can assemble into MB clusters such as {Mo_154_}^24^ and {Mo_176_},^27,28^ bent {Mo_8_-b} building blocks need to corporate with normal {Mo_8_} building blocks to accomplish high nuclearity MB constructions. We propose that the ratio of {Mo_8_-b} to {Mo_8_} is not random but can only be of some defined values. To realize the proposed structures, careful selection of supporting and bridging units is required.

To investigate the redox properties of the four new MB clusters produced in this study, cyclic voltammetry (CV) measurements were conducted in the solid state using a three-electrode setup in an aqueous solution saturated with N_2_. First, the redox behavior was investigated under various pH levels, with a specific focus on their chemical stability. Figure S10 displays the CVs of {Mo_54_} **2** in aqueous electrolytes at different pH values (pH 0 for a 0.5 M H_2_SO_4_ solution, pH 1 for 0.05 M H_2_SO_4_, and pH 2.5 for a H_2_SO_4_/Na_2_SO_4_ buffer solution). Under strong acidic media (pH 0), the material shows poor redox activity and low reversibility. At pH 1, the POM anion is stable and the five-step redox processes are clearly visible, which corresponds to the reduction of the Mo^VI^ centers.^60,61^ Increasing the pH to 2.5 results in a shift of the cathodic potentials to more negative values and a decrease in the peak current. These results indicate that the redox processes require the involvement of protons.

**Figure 5:**
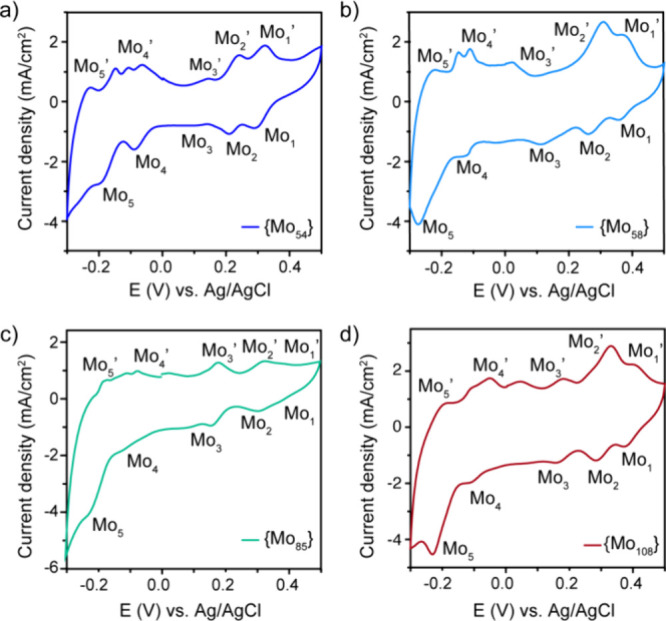
Cyclic voltammograms at pH 1. (a) {Mo_54_}, (b) {Mo_58_}, (c) {Mo_85_}, and (d) {Mo_108_}. Scan rate: 50 mV s^–1^.

Three reversible redox processes in the region of 0–0.5 V can be observed and are symbolized as Mo_1_, Mo_2_, and Mo_3_, as seen in Figure 5 and Figure S11. These peaks in the positive region can be seen more clearly in Figure S12, in a smaller potential window (from 0 to 0.5 V). Both cathodic (*E*_pc_) and anodic (*E*_pa_) peak potentials are very similar for all of the clusters (Table S14). Also, the peak-to-peak separations (Δ*E*_p_) vary between 0.02 and 0.04 V, showing a fast, reversible electron transfer process of the four redox-active compounds. When increasing the potential window, two more redox processes appear, named Mo_4_ and Mo_5_. The Mo_4_ process consists of one cathodic peak and two/three anodic peaks in negative potential. The redox peaks (Mo_1_ to Mo_5_) observed in the CV measurements were assigned to the five successive molybdenum redox processes (Mo(VI) → Mo(V)). It is important to emphasize that the distinct redox properties of Mo_1_ compared to Mo_5_ are strongly influenced by the structural and electronic features of the POM framework. Factors such as the differences in building blocks and their arrangement, the charge distribution within the POM, and interactions with counterions or the surrounding electrolyte contribute to the variation in redox potentials. To fully resolve the differences between Mo_1_ and Mo_5_ would require detailed complementary studies, such as spectroelectrochemistry, computational modeling, or X-ray absorption spectroscopy.

Additionally, electrochemical impedance spectroscopy (EIS) measurements in Figure S13 and Table S15 show that all of the clusters possess low interfacial resistance (Rr) (which is indicative of the fast charge transfer at the interface of the semiconductor and electrolyte). All these measurements confirm that these large molybdenum wheels favor charge transfer as shown by CV and impedance measurements.

## Conclusions

In conclusion, we have discovered and characterized a family of closed shell, highly compressed, MB type polyoxomolybdates. This study provides defined experimental control regarding conditions for the synthesis of complex inorganic clusters, where the resultant POMs have been characterized by SCXRD and electrochemical techniques. Our findings show that the geometry of giant Mo POMs can be controlled by incorporating different supporting/templating groups, such as SO_3_^2–^ and SO_4_^2–^; it was observed that geometric control of the cluster architecture can be obtained by utilizing templates adopting a range of geometries, by giving rise to different building blocks, such as the distorted bent pentagonal {Mo_6_} units. These units have displayed the capacity to give rise to an ensemble of new architectures undergoing a self-assembly process to form asymmetric MB POMs. With fine control of the synthetic parameters, we have produced four new clusters displaying unique frameworks of the MB backbone, exhibiting pseudo termination points by direct bonding of a pentagon peak in the backbone, as well as featuring unprecedented bridging unit {Mo_5_} extended from corner sharing {Mo_2_-c}. Additionally, we have shown electrochemical characterization in the solid state of the new structures reported in this work, revealing the Mo redox processes in these complex architectures. The highly compressed nature of these structures combined with the new synthetic approach means that these structures might be useful in developing new applications of these clusters as electronic materials and e-beam resists.

## Supporting Information Available

Figures S1–S13, Tables S1–S15, experimental details, synthetic procedures, SCXRD refinement details, formula determination, bond valence sum, thermogravimetric analysis, EDX, and electrochemical properties (PDF)
