# Supplementary material for: Compression of Molybdenum Blue Polyoxometalate Cluster Rings
Source: J Am Chem Soc. 2025 Mar 18;147(12):10579–86. doi: 10.1021/jacs.5c00187 (PMC11951155; doi:10.1021/jacs.5c00187)
Supplement: Supplementary file 1 — ja5c00187_si_001.pdf [file ja5c00187_si_001.pdf]

Supplementary Information

Compression of Molybdenum Blue Polyoxometalate Cluster Rings

Vishal Lakhanpal, Melanie Guillén-Soler, Laia Vilà-Nadal, De-Liang Long*, and Leroy Cronin*

School of Chemistry, University of Glasgow, Glasgow, G12 8QQ, United Kingdom.

Email: deliang.long@glasgow.ac.uk; lee.cronin@glasgow.ac.uk

Contents

1. Materials and Instrumentation 2

   Elemental Analyses 2

   Single Crystal X-Ray Diffraction 2

   pH Measurements 2

   Thermogravimetric Analysis 2

   Electrochemical Measurements 2

2. Synthesis Experimental 4

   Compound 1: $Na_2H_4[Mo^{V}_{12}Mo^{VI}_{46}H_{36}O_{180}(SO_4)_9](H_2O)_{35}$ $\{Mo_{58}\}$ 4

   Compound 2: $Na_4(NH_4)_5[Mo^{V}_{12}Mo^{VI}_{42}H_{33}O_{174}(SO_3)_3](H_2O)_{50}$ $\{Mo_{54}\}$ 4

   Compound 3: $Na_4H_{12}[Mo^{IV}_6Mo^{V}_{18}Mo^{VI}_{61}H_{48}O_{267}S_2(SO_3)_3](H_2O)_{85}$ $\{Mo_{85}\}$ 4

   Compound 4: $(NH_4)_{14}[Mo^{V}_{24}Mo^{VI}_{84}H_{78}O_{358}](H_2O)_{95}$ $\{Mo_{108}\}$ 5

3. Formula Determination 6

4. Crystallographic Data and Structure Refinement Details 7

5. Bond Valence Sums 11

   $\{Mo_{58}\}$ 11

   $\{Mo_{54}\}$ 12

   $\{Mo_{85}\}$ 14

   $\{Mo_{108}\}$ 15

6. Thermogravimetric Analysis (TGA) 17

7. EDX 19

8. Structure Comparisons 21

9. Electrochemical Measurement Results 27

1. Materials and Instrumentation

All reagents and solvents were purchased from commercial sources (Sigma Aldrich) and used as received.

Elemental Analyses

Elemental analyses for Mo, S, and Na were performed on the TESCAN CLARA scanning electron microscope using the Oxford Instruments UltimMax 65 EDS system coupled with the Aztec live interface. Samples were dried thoroughly to avoid solvent contamination of the system, therefore the theoretical contents of Mo and other elements are calculated from formula desolvated. Analysis for C, H and N content was performed on an Exeter CE-440 Elemental Analyser.

Single Crystal X-Ray Diffraction

A suitable single crystal was selected and mounted onto a rubber loop on goniometer using Fomblin or Parabar oil. Single-crystal datasets and unit cells for the relevant compounds were collected at 150(2) K on a Rigaku XtaLAB Synergy R HyPix-Arc diffractometer equipped with a graphite monochromator (λ (MoKα) = 0.71073 Å) on a microfocus X-ray source of rotating anode (50 kV, 24 mA). Data collection and reduction were performed using CrysAlisPro software package. Structure solution and refinement were carried out by SHELXT-2019/3[1] and SHELXL-2019/3[2] using Olex2[3] and finalized using WinGX.[4] Most of the non-hydrogen atoms (including those disordered) were anisotropically refined. Corrections for incident and diffracted beam absorption effects were applied using analytical numeric absorption correction on multifaceted crystal models. The X-ray crystallographic data in this manuscript have been deposited at the Crystallographic Data Centres with CCDC numbers 2381428-2381430 and 2410195. The data can be obtained free of charge from Cambridge Crystallographic Data Centre service www.ccdc.cam.ac.uk/structures with deposition number.

pH Measurements

Measurements were taken on a Hanna Instruments HI 9025C microcomputer pH meter, with a BCH combination pH electrode (309-1065) and HI 7669/2W temperature probe.

Thermogravimetric Analysis

Analysis for characterisation was performed on a TA Instruments Q 500 Thermogravimetric Analyser under air flow with a heating rate of 10 °C min^{-1} up to 1000 °C. Analysis for solution studies were performed on TA Discovery TGA 550 where ~5 mg solid samples were loaded onto a platinum pan, the weight-temperature changes were recorded under N_2 protection. Oven: Samples were heated in an LTE OP60-UF oven equipped with fan circulation. (TBC).

Electrochemical Measurements

Working electrode preparation

To prepare the working electrode, a dispersion of 2 mg each of the samples and 2 mg of carbon black (Vulcan X-72) in 2 mL of water was used as an ink. Thereafter, 10 µL of the obtained highly dispersed catalyst ink was dropped onto a mirror polished glassy carbon electrode (GCE) of 0.07 cm^2 of geometric surface area. Subsequently, 10 uL of Nafion solution (5 wt%) was added and the electrode was dried under ambient conditions.

Electrochemical characterization set-up

An Autolab potentiostat PGSTAT204 was used to measure the electrochemical characterization for all the clusters. All the electrochemical measurements were conducted in a three-electrode set-up, with a GCE, Ag/AgCl (3M KCl) and a platinum wire as working, reference and counter electrode, respectively. The measurements were carried out under N_2 atmosphere in an aqueous electrolyte solution as described.

Electrochemical measurements

Cyclic Voltammetry (CV) measurements under N_2-saturated aqueous solution were performed to characterize the materials. Current densities were normalized to the electrode geometrical area.

Impedance measurements

The cell resistance was measured immediately before ORR measurements taking the impedance spectra from 32 to 0.1 kHz and a voltage perturbation of 10 mV. The real part of the resistance at 1 kHz was taken as the cell resistance and was used to obtain the IR-free potential of the working electrode. The used circuit model was Rs(RrCdl), including solution resistance (Rs), interfacial resistance between semiconductor electrode and electrolyte (Rr), and constant phase element (Cdl), respectively. The Nyquist plots were then fitted by Zview software using the proposed equivalent circuit model.

2. Synthesis Experimental

Compound 1: $Na_2H_4[Mo^{V}_{12}Mo^{VI}_{46}H_{36}O_{180}(SO_4)_9](H_2O)_{35}$ {Mo_{58}}

To a 100 mL Erlenmeyer flask equipped with a magnetic stirrer bar, $(NH_4)_6Mo_7O_{24}\cdot4H_2O$ (8 g, 6.5 mmol) was dissolved in 40 mL of H_2O. The mixture was acidified via dropwise addition of 1.5 mL of 6 M H_2SO_4. Following this, $Na_2S_2O_4$ (1.48 g, 8.5 mmol) and $N_2H_4\cdot2HCl$ (1 g, 9.5 mmol) were separately dissolved in 20 mL of H_2O and introduced to solution under constant stirring. The pH is then decreased to 0.15 by dropwise addition of 6 M H_2SO_4. The solution is allowed to stir for one hour before being transferred to Teflon-lined stainless steel containers (21 mL mixture for each container, container volume 43 mL). The containers were kept at a constant 140 ^{o}C for 4 days before being allowed to cool to room temperature in the switched off oven. Samples were left undisturbed for 4 days before thin blue rods of compound **1** formed. Yield: 0.20g (2.7% based on Mo).

Compound 2: $Na_4(NH_4)_5[Mo^{V}_{12}Mo^{VI}_{42}H_{33}O_{174}(SO_3)_3](H_2O)_{50}$ {Mo_{54}}

To a 100mL Erlenmeyer flask equipped with a magnetic stirrer bar, $(NH_4)_6Mo_7O_{24}\cdot4H_2O$ (8 g, 6.5 mmol) was dissolved in 40 mL of H_2O. The mixture was acidified via dropwise addition of 1.5 mL of 6 M H_2SO_4. Following this, $Na_2S_2O_4$ (1.48 g, 8.5 mmol) and $N_2H_4\cdot2HCl$ (1 g, 9.5 mmol) were separately dissolved in 20 mL of H_2O and introduced to solution under constant stirring. The mixture was allowed to stir for 1 hour during which the colour transitions from dark green to dark blue. The pH was then adjusted to 2.8, from approximately 3.1, by dropwise addition of 6M H_2SO_4. The sample is left to stir for a further hour before undergoing gravity filtration. Following a further 12 hours the sample was filtered before being left for 2 weeks undisturbed for crystal growth. Small blue prismatic crystals of **2** were formed alongside larger blue hexagonal crystals of {Mo_{18}} as side-product, which we determined single crystal structure and its cluster framework is similar to $[Mo_{18}O_{54}(SO_3)_2]^{4-}$.[5] Detailed results of this {Mo_{18}} side-product will be published elsewhere. Yield: 0.72g (9.2% based on Mo). Attempts at purifying {Mo_{54}} were made using a fine nylon mesh, however it was observed that the mesh sizes tested would remove large crystals of {Mo_{18}} but smaller crystals of {Mo_{18}} and {Mo_{54}} were of similar sizes, thus posing challenges to selectively remove the {Mo_{18}}.

Compound 3: $Na_4H_{12}[Mo^{IV}_6Mo^{V}_{18}Mo^{VI}_{61}H_{48}O_{267}S_2(SO_3)_3](H_2O)_{85}$ {Mo_{85}}

To a 100mL Erlenmeyer flask equipped with a magnetic stirrer bar, $(NH_4)_6Mo_7O_{24}\cdot4H_2O$ (8 g, 6.5 mmol) was dissolved in 40 mL of H_2O. The mixture was acidified via dropwise addition of 1.5 mL of 6 M H_2SO_4. Then $Na_2S_2O_4$ (1.48 g, 8.5 mmol) and $N_2H_4\cdot2HCl$ (1 g, 9.5 mmol) were separately dissolved in 20 mL of H_2O and introduced to solution under constant stirring. The pH was then adjusted to 2.5, from approximately 2.8, by dropwise addition of 6 M H_2SO_4. The solution is allowed to stir for one hour before being transferred to Teflon-lined stainless-steel containers (21 mL mixture for each container, container volume 43 mL). The containers were kept at a constant 140 ^{o}C for 3 days before being allowed to cool to room temperature in the switched off oven. Samples were left undisturbed for 2 days before large blue-plate crystals of compound **3** formed. Yield: 0.24g (3.1% based on Mo). In initial synthesis experiment, TiO_2 (1.6 g, 20 mmol) was added before adding $Na_2S_2O_4$ and $N_2H_4\cdot2HCl$ reducing agents but late optimised experiment found that {Mo_{85}} can be obtained without TiO2 involvement.

Compound 4: $(NH_4)_{14}[Mo^{V}_{24}Mo^{VI}_{84}H_{78}O_{358}](H_2O)_{95}$ $\{Mo_{108}\}$

To a glass vial equipped with a magnetic stirrer bar, $(NH_4)_6Mo_7O_{24}\cdot 4H_2O$ (617 mg, 0.5 mmol) was dissolved in 3 mL of H_2O. Following this, $N_2H_4\cdot 2HCl$ (50 mg, 0.5 mmol) was dissolved in 0.5 ml H_2O and introduced to the molybdate solution, where it was then left to stir for 10 minutes. Then $Li_2B_4O_7$ (50 mg, 0.3 mmol) was dissolved in 0.5 mL of H_2O and introduced to the molybdate solution under constant stirring, where it was left to stir for 10 minutes. The mixture was then acidified to pH 1.05, via addition of 500 µL of 6 M HCl. The final solution was then left to stir for 1 hour before being transferred to 10 mL vials, capped with a metal cap equipped with a diaphragm. It was heated at 120 ^{o}C for 3 days after which large blue cubic crystals of **4** formed. Yield 200 mg (34.1% based on Mo).

3. Formula Determination

To analyse mix-valence Mo clusters a number of analytical techniques should be employed, as appropriate bond valence sums (BVS), redox titrations, thermogravimetric analysis (TGA), single-crystal X-ray diffraction analysis (SCXRD), elemental analyses including energy dispersive spectroscopy and CHN analysis were utilized to determine the formulae of compounds **1** to **4**. Compound **4** was selected as an idealized example of how formulae determination was performed.

First SCXRD was performed on a single crystal to obtain information on the approximate formula and bond lengths of compound **4**. Following this, BVS were performed on all Mo and O centres, suggesting that **4** is a 24-electron reduced (Redox titration showed 27) oblate spheroid, where it also possesses 78 protons. Due to the delocalisation of electrons they cannot be discretely distributed but instead using BVS we can identify that bridging positions, $\{Mo_1\}$, $\{Mo_3\}$ and $\{Mo_5\}$, have greater probability of retaining the delocalised electrons.

EDS indicated that the cluster of **4**, by weight %, contains 64.8% Mo, 33.5% O and 1.63% N. Accounting for TGA, from r.t. to 200 ^{o}C, the cluster displayed about 9.5% weight loss indicating the loss of 95 guest H_2O molecules.

From the above analyses we can suggest that the final formula of compound **4** is approximately $(NH_4)_{14}[Mo_{108}H_{78}O_{358}]\cdot 95H_2O$.

The formulae for compounds **1** to **3** were determined similarly to **4**. The structural contents were identified using EDS, where single crystals were mounted and analysed. These were compared to the structural refinements which were first freely refined and then fixed. The number of protons on clusters were assigned using BVS and the number of solvent water molecules were tentatively assigned via bulk TGA analysis.

4. Crystallographic Data and Structure Refinement Details

Table S1. Crystal data and structure refinement for $Na_2H_4[Mo_{58}H_{36}O_{180}(SO_4)_9](H_2O)_{35}$ (**1**)[†]

Empirical formula	$H_{110}Mo_{58}Na_2O_{251}S_9$	
Formula weight	10026.29	
Temperature	150(2) K	
Wavelength	0.71073 Å	
Crystal system	Orthorhombic	
Space group	*Cmcm*	
Unit cell dimensions	a = 15.7155(2) Å	α = 90°.
	b = 35.8831(4) Å	β = 90°.
	c = 38.6564(4) Å	γ = 90°.
Volume	21799.1(4) $Å^3$	
Z	4	
Density (calculated)	3.055 Mg/m^3	
Absorption coefficient	3.436 mm^{-1}	
F(000)	18880	
Crystal size	0.100 x 0.010 x 0.010 mm^3	
Theta range for data collection	2.270 to 26.000°.	
Index ranges	-19<=h<=19, -44<=k<=44, -47<=l<=47	
Reflections collected	226211	
Independent reflections	11287 [R(int) = 0.0707]	
Completeness to theta = 25.242°	99.8 %	
Refinement method	Full-matrix least-squares on F^2	
Data / restraints / parameters	11287 / 36 / 782	
Goodness-of-fit on F^2	1.075	
Final R indices [I>2σ(I)]	R1 = 0.0375, wR2 = 0.0932	
R indices (all data)	R1 = 0.0457, wR2 = 0.0973	
Largest diff. peak and hole	1.83 and -1.59 e.$Å^{-3}$	

[†]Notes: one quarter of the {Mo_{58}} cluster was found in the asymmetric unit. SO_4 templates on cluster backbone positions were well-defined. A SO_4 ligand (involving S4) was found disordered inside the cluster. A SO_4 ligand (involving S5) was identified on the cluster edge in the gaps between pentagonal building blocks. Another low occupied SO_4 ligand (involving S6) on similar positions of cluster edge between pentagonal building blocks was found disordered with the bridging {Mo_2} unit (involving Mo7). Total number of SO_4 ligands was assessed from structure refinement and elemental analysis. O, OH or H_2O ligands on cluster were identified by BVS results of oxygen atoms. No H atoms were added to O atoms as it is a heavy metal cluster compound. SQUEEZE procedure[6] (from PLATON) was applied to calculate the void space and the electron counts in solvent area. Crystallography formula was assessed from structure refinement, SQUEEZE procedure results and elemental analysis.

Table S2. Crystal data and structure refinement for $Na_4(NH_4)_5[Mo_{54}H_{33}O_{174}(SO_3)_3](H_2O)_{50}$ (**2**) ‡

Empirical formula	$H_{153}Mo_{54}N_5Na_4O_{233}S_3$	
Formula weight	9321.16	
Temperature	150(2) K	
Wavelength	0.71073 Å	
Crystal system	Orthorhombic	
Space group	*Pnma*	
Unit cell dimensions	a = 26.4475(3) Å	α = 90°.
	b = 32.4012(3) Å	β = 90°.
	c = 24.02510(10) Å	γ = 90°.
Volume	20587.8(3) Å^3	
Z	4	
Density (calculated)	3.007 Mg/m^3	
Absorption coefficient	3.338 mm^{-1}	
F(000)	17648	
Crystal size	0.072 x 0.043 x 0.016 mm^3	
Theta range for data collection	2.291 to 26.000°.	
Index ranges	-32<=h<=32, -39<=k<=39, -29<=l<=29	
Reflections collected	667300	
Independent reflections	20604 [R(int) = 0.1131]	
Completeness to theta = 25.242°	99.9 %	
Refinement method	Full-matrix least-squares on F^2	
Data / restraints / parameters	20604 / 114 / 1466	
Goodness-of-fit on F^2	1.035	
Final R indices [I>2σ(I)]	R1 = 0.0394, wR2 = 0.0930	
R indices (all data)	R1 = 0.0558, wR2 = 0.1003	
Largest diff. peak and hole	2.58 and -2.71 e.Å^{-3}	

‡Notes: half {Mo_{54}} cluster was found in the asymmetric unit. Each SO_3 template was found disordered over nearby two positions with each S site having half occupancies (see S1 and S1' for example). Sodium atoms were identified by using criteria Na-O bond distances being around 2.4 Å. N atoms of NH_4^+ in solvent area were assigned to sites with N…O distances to neighbouring oxo or water larger than 2.80 Å. O, OH or H_2O ligands were identified by BVS of oxygen atoms on cluster. No H atoms were added to O atoms as it is a heavy metal cluster compound. Crystallography formula was assessed from structure refinement and elemental analysis.

Table S3. Crystal data and structure refinement for $Na_4H_{12}[Mo_{85}H_{48}O_{267}S_2(SO_3)_3](H_2O)_{85}$ (**3**)[#]

Empirical formula	$H_{218}Mo_{85}Na_6O_{361}S_5$	
Formula weight	14414.99	
Temperature	150(2) K	
Wavelength	0.71073 Å	
Crystal system	Orthorhombic	
Space group	*Cmcm*	
Unit cell dimensions	a = 43.6494(3) Å	α = 90°.
	b = 29.8465(4) Å	β = 90°.
	c = 27.1318(2) Å	γ = 90°.
Volume	35346.8(6) Å^3	
Z	4	
Density (calculated)	2.709 Mg/m^3	
Absorption coefficient	3.055 mm^{-1}	
F(000)	27248	
Crystal size	0.110 x 0.036 x 0.010 mm^3	
Theta range for data collection	2.395 to 25.999°.	
Index ranges	-53<=h<=53, -36<=k<=36, -33<=l<=33	
Reflections collected	574432	
Independent reflections	18003 [R(int) = 0.1002]	
Completeness to theta = 25.242°	99.9 %	
Refinement method	Full-matrix least-squares on F^2	
Data / restraints / parameters	18003 / 36 / 1111	
Goodness-of-fit on F^2	1.042	
Final R indices [I>2σ(I)]	R1 = 0.0486, wR2 = 0.1282	
R indices (all data)	R1 = 0.0656, wR2 = 0.1385	
Largest diff. peak and hole	2.92 and -2.08 e.Å^{-3}	

[#]Notes: one quarter of the {Mo_{85}} cluster was found in the asymmetric unit. The tetrahedrally coordinated Mo28 was found to have half occupancy and share site possibly with protons. A {Mo_3S} moiety (involving Mo1 and Mo2) was identified on cluster with minor rotational disorders (Mo1' and Mo2'). Another {Mo_3S} moiety site (Mo26 and Mo27) of very low occupancy (therefore not included in formula) was identified on cluster back (near Mo3). A SO_3 ligand (involving S3) on cluster surface and a SO_3 ligand (involving S2) inside cluster were found each disordered over nearby two positions with every S site having half occupancy. Total number of SO_3 ligands was assessed from structure refinement and elemental analysis. O, OH or H_2O ligands on cluster were identified by BVS results of oxygen atoms. No H atoms were added to O atoms as it is a heavy metal cluster compound. SQUEEZE procedure (from PLATON) was applied to calculate the void space and the electron counts in solvent area. Number of solvent water molecules for crystallography formula was assessed from structure refinement and SQUEEZE procedure results.

Table S4. Crystal data and structure refinement for $(NH_4)_{14}[Mo_{108}H_{78}O_{358}](H_2O)_{95}$ {Mo_{108}} (**4**)[§]

Empirical formula	$H_{324}Mo_{108}N_{14}O_{453}$	
Formula weight	18132.23	
Temperature	150(2) K	
Wavelength	0.71073 Å	
Crystal system	Monoclinic	
Space group	*C*2/*m*	
Unit cell dimensions	a = 25.7745(2) Å	α = 90°.
	b = 28.1300(2) Å	β = 103.297(1)°.
	c = 28.2188(2) Å	γ = 90°.
Volume	19911.2(3) Å^3	
Z	2	
Density (calculated)	3.024 Mg/m^3	
Absorption coefficient	3.407 mm^{-1}	
F(000)	17164	
Crystal size	0.110 x 0.060 x 0.040 mm^3	
Theta range for data collection	2.176 to 26.000°.	
Index ranges	-31<=h<=31, -34<=k<=34, -34<=l<=34	
Reflections collected	181872	
Independent reflections	19977 [R(int) = 0.0275]	
Completeness to theta = 25.242°	99.9 %	
Absorption correction	Gaussian	
Max. and min. transmission	1.000 and 0.709	
Refinement method	Full-matrix least-squares on F^2	
Data / restraints / parameters	19977 / 54 / 1284	
Goodness-of-fit on F^2	1.025	
Final R indices [I>2σ(I)]	R1 = 0.0241, wR2 = 0.0653	
R indices (all data)	R1 = 0.0282, wR2 = 0.0671	
Largest diff. peak and hole	2.58 and -2.59 e.Å^{-3}	

[§]Notes: One quarter of the {Mo_{108}} cluster was found in the asymmetric unit. Sodium sits were identified by using criteria Na-O bond distances being around 2.4 Å. N atoms of NH4+ in solvent area were assigned to sites with N…O distances to neighbouring oxo or water sites larger than 2.80 Å. O, OH or H_2O ligands on cluster were identified by BVS of oxygen atoms. No H atoms were added to O atoms as it is a heavy metal cluster compound. Crystallography formula was assessed from structure refinement and elemental analysis. At the sites Mo31 and Mo32, there are some electron density peaks higher than usual solvent area. They were considered to be Mo-Mo dimer with about 5% occupancy and were not counted in formula due to too low occupancy.

5. Bond Valence Sums

Bond valence calculations were carried out for each bond using the measured bond distance (R) and parameters R0 and B, where bond valence = EXP((R0-R)/B). The individual bond valences were then summed to determine the BVS. R0 and B were taken from Gagne & Hawthorne[7] and Liu & Thorp.[8] Mo(6+), Mo(5+) and Mo(4+) centres were calculated using different empirical values of R0 and B where are appropriate. Otherwise R0 and B values of Mo(6+)-O(2-) were used.

Parameter ist:

Bond	R0	B
Mo(6+)-O(2-)	1.903	0.349
Mo(5+)-O(2-)	1.888	0.314
Mo(4+)-O(2-)	1.834	0.404
Mo(4+)-S(2-)	2.235	0.370

{Mo_{58}}

Mo BVS

Bond		R	BVS	Bond		R	BVS	Bond		R	BVS	Bond		R	BVS
Mo1	O1	1.69	1.841	Mo2	O5	1.67	1.950	Mo3	O9	1.67	1.950	Mo4	O15	1.67	1.950
Mo1	O17	1.993	0.773	Mo2	O7	1.841	1.194	Mo3	O10	1.899	1.012	Mo4	O16	1.802	1.336
Mo1	O17	1.993	0.773	Mo2	O6	1.895	1.023	Mo3	O7	1.946	0.884	Mo4	O13	1.831	1.229
Mo1	O4	2.02	0.715	Mo2	O4	2.008	0.740	Mo3	O13	1.964	0.840	Mo4	O17	2.054	0.649
Mo1	O4	2.021	0.713	Mo2	O3	2.054	0.649	Mo3	O12	1.983	0.795	Mo4	O4	2.065	0.629
Mo1	O3	2.071	0.618	Mo2	O8	2.437	0.217	Mo3	O11	2.439	0.215	Mo4	O14	2.483	0.190
Mo1	O2	2.252	0.368												
			5.800				5.773				5.695				5.982
Mo5	O18	1.686	1.862	Mo6	O22	1.663	1.989	Mo7	O25	1.646	2.088	Mo8	O29	1.671	1.944
Mo5	O20	1.829	1.236	Mo6	O24	1.968	0.830	Mo7	O27	1.856	1.144	Mo8	O30	1.902	1.003
Mo5	O20	1.829	1.236	Mo6	O16	1.978	0.807	Mo7	O28	1.87	1.099	Mo8	O32	1.934	0.915
Mo5	O17	2.041	0.673	Mo6	O20	1.979	0.804	Mo7	O12	1.904	0.997	Mo8	O33	1.961	0.847
Mo5	O17	2.041	0.673	Mo6	O23	2.002	0.753	Mo7	O26	2.139	0.509	Mo8	O28	1.983	0.795
Mo5	O19	2.36	0.270	Mo6	O21	2.278	0.341	Mo7	O63	2.52	0.171	Mo8	O31	2.405	0.237
			5.951				5.525				6.008				5.741
Mo9	O35	1.681	1.889	Mo10	O44	1.68	1.895	Mo11	O41	1.662	1.995	Mo12	O39	1.702	1.779
Mo9	O24	1.804	1.328	Mo10	O32	1.848	1.171	Mo11	O37	1.994	0.770	Mo12	O62	1.819	1.272
Mo9	O33	1.828	1.240	Mo10	O45	1.898	1.014	Mo11	O40	2.012	0.732	Mo12	O23	1.825	1.250
Mo9	O37	2.047	0.662	Mo10	O36	2.009	0.738	Mo11	O46	2.019	0.717	Mo12	O37	2.051	0.654
Mo9	O36	2.071	0.618	Mo10	O46	2.045	0.666	Mo11	O51	2.032	0.691	Mo12	O40	2.053	0.651
Mo9	O34	2.468	0.198	Mo10	O43	2.435	0.218	Mo11	O36	2.037	0.681	Mo12	O38	2.343	0.283
								Mo11	O42	2.301	0.320				
			5.935				5.701				5.906				5.890
Mo13	O47	1.676	1.916	Mo14	O53	1.67	1.950	Mo15	O58	1.682	1.884	Mo16	O60	1.679	1.900
Mo13	O49	1.86	1.131	Mo14	O49	1.899	1.012	Mo15	O59	1.808	1.313	Mo16	O62	1.992	0.775
Mo13	O48	1.896	1.020	Mo14	O54	1.907	0.989	Mo15	O56	1.848	1.171	Mo16	O62	1.992	0.775
Mo13	O51	2.01	0.736	Mo14	O56	1.934	0.915	Mo15	O40	2.026	0.703	Mo16	O59	1.997	0.764
Mo13	O46	2.026	0.703	Mo14	O55	2.059	0.640	Mo15	O51	2.094	0.579	Mo16	O59	1.998	0.762
Mo13	O50	2.462	0.202	Mo14	O52	2.403	0.239	Mo15	O57	2.473	0.195	Mo16	O61	2.239	0.382
			5.708				5.743				5.844				5.357

{Mo_{54}}

Mo BVS

Bond		R	BVS	Bond		R	BVS	Bond		R	BVS	Bond		R	BVS
Mo1	O65	1.683	1.878	Mo2	O13	1.688	1.852	Mo3	O67	1.674	1.927	Mo4	O86	1.667	1.966
Mo1	O53	2.01	0.736	Mo2	O60	2.003	0.751	Mo3	O9	1.991	0.777	Mo4	O30	1.982	0.797
Mo1	O27	2.014	0.728	Mo2	O60	2.003	0.751	Mo3	O19	2.003	0.751	Mo4	O82	2.018	0.719
Mo1	O26	2.015	0.725	Mo2	O72	2.014	0.728	Mo3	O32	2.007	0.742	Mo4	O82	2.018	0.719
Mo1	O7	2.017	0.721	Mo2	O72	2.014	0.728	Mo3	O22	2.057	0.643	Mo4	O66	2.06	0.638
Mo1	O29	2.081	0.600	Mo2	O40	2.08	0.602	Mo3	O69	2.06	0.638	Mo4	O66	2.06	0.638
Mo1	O38	2.203	0.423	Mo2	O6	2.195	0.433	Mo3	O71	2.241	0.380	Mo4	O59	2.241	0.380
			5.812				5.844				5.858				5.858
Mo5	O37	1.668	1.961	Mo6	O58	1.683	1.878	Mo7	O62	1.677	1.911	Mo8	O61	1.664	1.983
Mo5	O45	1.955	0.862	Mo6	O17	1.823	1.258	Mo7	O15	1.827	1.243	Mo8	O12	1.823	1.258
Mo5	O45	1.955	0.862	Mo6	O17	1.823	1.258	Mo7	O35	1.87	1.099	Mo8	O4	1.869	1.102
Mo5	O18	2.008	0.740	Mo6	O66	2.065	0.629	Mo7	O26	2.019	0.717	Mo8	O72	2.035	0.685
Mo5	O18	2.008	0.740	Mo6	O66	2.065	0.629	Mo7	O29	2.072	0.616	Mo8	O40	2.062	0.634
Mo5	O55	2.389	0.248	Mo6	O59	2.493	0.184	Mo7	O77	2.345	0.282	Mo8	O21	2.361	0.269
			5.413				5.835				5.869				5.932
Mo9	O48	1.67	1.950	Mo10	O33	1.68	1.895	Mo11	O70	1.693	1.825	Mo12	O20	1.688	1.852
Mo9	O44	1.842	1.191	Mo10	O12	1.924	0.942	Mo11	O36	1.852	1.157	Mo12	O51	1.826	1.247
Mo9	O11	1.879	1.071	Mo10	O36	1.95	0.874	Mo11	O2	1.857	1.141	Mo12	O51	1.826	1.247
Mo9	O27	2.032	0.691	Mo10	O14	1.954	0.864	Mo11	O19	2.003	0.751	Mo12	O60	2.075	0.611
Mo9	O29	2.08	0.602	Mo10	O24	2.013	0.730	Mo11	O69	2.051	0.654	Mo12	O60	2.075	0.611
Mo9	O76	2.322	0.301	Mo10	O79	2.322	0.301	Mo11	O79	2.395	0.244	Mo12	O3	2.395	0.244
			5.806				5.605				5.773				5.811
Mo13	O31	1.665	1.978	Mo14	O49	1.683	1.878	Mo15	O75	1.689	1.846	Mo16	O88	1.684	1.873
Mo13	O15	1.947	0.882	Mo14	O34	1.902	1.003	Mo15	O43	1.815	1.287	Mo16	O44	1.918	0.958
Mo13	O68	1.952	0.869	Mo14	O11	1.96	0.849	Mo15	O24	1.82	1.268	Mo16	O39	1.944	0.889
Mo13	O54	1.96	0.849	Mo14	O81	1.974	0.816	Mo15	O22	2.061	0.636	Mo16	O25	1.954	0.864
Mo13	O43	2.008	0.740	Mo14	O73	1.998	0.762	Mo15	O69	2.068	0.623	Mo16	O17	2.001	0.755
Mo13	O41	2.34	0.286	Mo14	O76	2.233	0.388	Mo15	O71	2.503	0.179	Mo16	O50	2.319	0.304
			5.604				5.697				5.840				5.643
Mo17	O78	1.673	1.933	Mo18	O8	1.696	1.810	Mo19	O46	1.684	1.873	Mo20	O96	1.636	2.149
Mo17	O5	1.956	0.859	Mo18	O10	1.819	1.272	Mo19	O25	1.847	1.174	Mo20	O64	1.825	1.250
Mo17	O28	1.959	0.852	Mo18	O18	1.833	1.222	Mo19	O34	1.855	1.147	Mo20	O93	1.953	0.867
Mo17	O51	2.001	0.755	Mo18	O53	2.069	0.621	Mo19	O82	1.999	0.760	Mo20	O89	2.052	0.653
Mo17	O10	2.009	0.738	Mo18	O7	2.074	0.613	Mo19	O66	2.06	0.638	Mo20	O9	2.077	0.607
Mo17	O85	2.402	0.239	Mo18	O52	2.375	0.259	Mo19	O50	2.409	0.235	Mo20	O19	2.152	0.490
			5.376				5.797				5.826				6.016
Mo21	O56	1.707	1.753	Mo22	O57	1.681	1.889	Mo23	O87	1.676	1.916	Mo24	O84	1.686	1.862
Mo21	O6	1.788	1.390	Mo22	O68	1.854	1.151	Mo23	O45	1.8	1.343	Mo24	O23	1.913	0.972
Mo21	O21	1.919	0.955	Mo22	O23	1.868	1.105	Mo23	O39	1.86	1.131	Mo24	O80	1.959	0.852
Mo21	O21	1.919	0.955	Mo22	O32	2.005	0.747	Mo23	O53	2.063	0.632	Mo24	O35	1.98	0.802
Mo21	O1	2.101	0.567	Mo22	O22	2.041	0.673	Mo23	O27	2.084	0.595	Mo24	O73	1.985	0.791
Mo21	O40	2.561	0.152	Mo22	O41	2.417	0.229	Mo23	O63	2.343	0.283	Mo24	O77	2.246	0.374
			5.773				5.795				5.902				5.653
Mo25	O90	1.664	1.983	Mo26	O74	1.685	1.868	Mo27	O83	1.705	1.764	Mo28	O16	1.675	1.922
Mo25	O28	1.812	1.298	Mo26	O5	1.815	1.287	Mo27	O38	1.792	1.374	Mo28	O2	1.898	1.014
Mo25	O54	1.859	1.134	Mo26	O14	1.858	1.138	Mo27	O77	1.925	0.939	Mo28	O4	1.962	0.844
Mo25	O7	2.056	0.645	Mo26	O60	2.05	0.656	Mo27	O76	1.929	0.928	Mo28	O64	1.969	0.828
Mo25	O26	2.097	0.574	Mo26	O72	2.078	0.606	Mo27	O73	2.098	0.572	Mo28	O1	1.994	0.770
Mo25	O42	2.336	0.289	Mo26	O47	2.35	0.278	Mo27	O29	2.542	0.160	Mo28	O21	2.227	0.395
			5.923				5.832				5.737				5.774

Mo29	O95	1.645	2.094	Mo30	O97	1.639	2.131
Mo29	O80	1.843	1.188	Mo30	O81	1.852	1.157
Mo29	O94	1.884	1.056	Mo30	O92	1.898	1.014
Mo29	O9	2.07	0.620	Mo30	O30	2.098	0.572
Mo29	O91	2.119	0.539	Mo30	O98	2.108	0.556
Mo29	O32	2.15	0.493	Mo30	O82	2.135	0.514
			5.989				5.945

{Mo$_{85}$}

Mo BVS

Bond		R	BVS	Bond		R	BVS	Bond		R	BVS	Bond		R	BVS
Mo1	O75	1.912	0.824	Mo2	O75	1.913	0.822	Mo3	O70	1.659	2.012	Mo4	O21	1.685	1.868
Mo1	O75	1.912	0.824	Mo2	O76	1.922	0.804	Mo3	O8	1.947	0.882	Mo4	O28	1.805	1.324
Mo1	O61	2.099	0.519	Mo2	O4	2.062	0.569	Mo3	O8	1.947	0.882	Mo4	O22	1.817	1.279
Mo1	O61	2.099	0.519	Mo2	O71	2.09	0.531	Mo3	O8	1.947	0.882	Mo4	O47	2.054	0.649
Mo1	O74	2.204	0.400	Mo2	O73	2.215	0.389	Mo3	O8	1.947	0.882	Mo4	O62	2.068	0.623
Mo1	S1	2.378	0.679	Mo2	S1	2.359	0.715	Mo3	O80	2.229	0.393	Mo4	O7	2.393	0.246
			3.766				3.831				5.931				5.989
Mo5	O44	1.683	1.878	Mo6	O34	1.678	1.905	Mo7	O55	1.693	1.825	Mo8	O37	1.674	1.927
Mo5	O46	1.839	1.201	Mo6	O12	1.843	1.188	Mo7	O57	1.851	1.161	Mo8	O47	2.004	0.749
Mo5	O64	1.866	1.112	Mo6	O31	1.86	1.131	Mo7	O38	1.874	1.087	Mo8	O62	2.005	0.747
Mo5	O54	2.009	0.738	Mo6	O52	2.023	0.709	Mo7	O49	2.015	0.725	Mo8	O49	2.02	0.715
Mo5	O30	2.054	0.649	Mo6	O59	2.114	0.546	Mo7	O59	2.092	0.582	Mo8	O52	2.024	0.707
Mo5	O51	2.442	0.213	Mo6	O27	2.347	0.280	Mo7	O36	2.304	0.317	Mo8	O59	2.079	0.604
												Mo8	O63	2.2	0.427
			5.792				5.760				5.697				5.876
Mo9	O42	1.68	1.895	Mo10	O14	1.686	1.862	Mo11	O3	1.683	1.878	Mo12	O39	1.675	1.922
Mo9	O25	1.811	1.302	Mo10	O6	1.849	1.167	Mo11	O67	1.77	1.464	Mo12	O23	1.999	0.760
Mo9	O25	1.811	1.302	Mo10	O33	1.85	1.164	Mo11	O51	1.96	0.849	Mo12	O23	1.999	0.760
Mo9	O11	2.053	0.651	Mo10	O23	2.02	0.715	Mo11	O51	1.96	0.849	Mo12	O1	1.999	0.760
Mo9	O11	2.053	0.651	Mo10	O11	2.037	0.681	Mo11	O5	2.094	0.579	Mo12	O11	2.044	0.668
Mo9	O29	2.678	0.109	Mo10	O56	2.371	0.262	Mo11	O30	2.524	0.169	Mo12	O11	2.045	0.666
												Mo12	O29	2.357	0.272
			5.908				5.851				5.788				5.806
Mo13	O41	1.659	2.012	Mo14	O43	1.689	1.846	Mo15	O61	1.752	1.541	Mo16	O48	1.684	1.873
Mo13	O9	1.965	0.837	Mo14	O63	1.798	1.351	Mo15	O10	1.788	1.390	Mo16	O12	1.916	0.963
Mo13	O9	1.965	0.837	Mo14	O27	1.898	1.014	Mo15	O19	1.995	0.768	Mo16	O6	1.938	0.905
Mo13	O28	1.986	0.788	Mo14	O36	1.928	0.931	Mo15	O16	2.005	0.747	Mo16	O2	1.964	0.840
Mo13	O28	1.986	0.788	Mo14	O53	2.156	0.484	Mo15	O1	2.064	0.630	Mo16	O25	2.044	0.668
Mo13	O45	2.346	0.281	Mo14	O59	2.47	0.197	Mo15	O23	2.1	0.569	Mo16	O56	2.274	0.345
			5.544				5.824				5.646				5.594
Mo17	O68	1.677	1.911	Mo18	O35	1.685	1.868	Mo19	O40	1.677	1.911	Mo20	O50	1.679	1.900
Mo17	O20	1.99	0.779	Mo18	O57	1.914	0.969	Mo19	O46	1.907	0.989	Mo20	O33	1.895	1.023
Mo17	O20	1.99	0.779	Mo18	O64	1.959	0.852	Mo19	O38	1.938	0.905	Mo20	O31	1.933	0.918
Mo17	O54	2.007	0.742	Mo18	O60	2.001	0.755	Mo19	O53	1.963	0.842	Mo20	O53	1.986	0.788
Mo17	O54	2.007	0.742	Mo18	O5	2.024	0.707	Mo19	O15	1.998	0.762	Mo20	O10	2.026	0.703
Mo17	O30	2.102	0.565	Mo18	O51	2.269	0.350	Mo19	O36	2.282	0.338	Mo20	O27	2.29	0.330
Mo17	O67	2.211	0.414												
			5.933				5.501				5.745				5.662
Mo21	O69	1.704	1.769	Mo22	O26	1.649	2.071	Mo23	O66	1.651	2.059	Mo24	O9	1.818	1.276
Mo21	O71	1.772	1.456	Mo22	O4	1.782	1.414	Mo23	O58	1.844	1.184	Mo24	O2	1.837	1.208
Mo21	O15	1.815	1.287	Mo22	O4	1.782	1.414	Mo23	O32	1.977	0.809	Mo24	O77	1.892	1.032
Mo21	O20	2.068	0.623	Mo22	O20	2.08	0.602	Mo23	O22	1.987	0.786	Mo24	O52	2.039	0.677
Mo21	O54	2.109	0.554	Mo22	O20	2.08	0.602	Mo23	O8	2.079	0.604	Mo24	O47	2.041	0.673
Mo21	O18	2.327	0.297	Mo22	O78	2.422	0.226	Mo23	O79	2.285	0.335	Mo24	O17	2.375	0.259
			5.985				6.330				5.777				5.125
Mo25	O24	1.68	1.895	Mo28	O56	1.674	1.927								
Mo25	O32	1.803	1.332	Mo28	O19	1.794	1.367								
Mo25	O60	1.829	1.236	Mo28	O29	1.852	1.157								
Mo25	O49	2.065	0.629	Mo28	O77	1.971	0.823								

Mo25	O62	2.065	0.629	Mo28	O23	2.932	0.052
Mo25	O13	2.32	0.303	Mo28	O27	2.911	0.056
			6.023				5.382

{Mo_{108}}

Mo BVS

Bond		R	BVS	Bond		R	BVS	Bond		R	BVS	Bond		R	BVS
Mo1	O48	1.679	1.901	Mo2	O32	1.699	1.795	Mo3	O15	1.678	1.908	Mo4	O14	1.679	1.899
Mo1	O23	1.900	1.009	Mo2	O40	1.790	1.382	Mo3	O37	1.908	0.986	Mo4	O55	1.832	1.225
Mo1	O33	1.947	0.881	Mo2	O38	1.838	1.204	Mo3	O7	1.947	0.881	Mo4	O7	1.858	1.139
Mo1	O4	1.980	0.802	Mo2	O3	2.053	0.651	Mo3	O42	1.981	0.800	Mo4	O1	2.021	0.714
Mo1	O38	2.001	0.755	Mo2	O10	2.074	0.613	Mo3	O4	1.990	0.780	Mo4	O2	2.048	0.660
Mo1	O49	2.267	0.353	Mo2	O29	2.314	0.308	Mo3	O26	2.307	0.314	Mo4	O26	2.422	0.226
			5.700				5.952				5.669				5.863
Mo5	O19	1.669	1.957	Mo6	O73	1.681	1.887	Mo7	O54	1.688	1.852	Mo8	O59	1.680	1.896
Mo5	O20	1.976	0.812	Mo6	O23	1.834	1.219	Mo7	O22	1.789	1.387	Mo8	O37	1.831	1.228
Mo5	O20	1.976	0.812	Mo6	O33	1.865	1.116	Mo7	O78	1.891	1.034	Mo8	O27	1.862	1.125
Mo5	O6	1.989	0.782	Mo6	O3	2.028	0.700	Mo7	O60	1.933	0.918	Mo8	O47	2.020	0.715
Mo5	O6	1.989	0.782	Mo6	O2	2.043	0.670	Mo7	O51	2.126	0.528	Mo8	O43	2.054	0.649
Mo5	O34	2.333	0.292	Mo6	O49	2.392	0.247	Mo7	O43	2.505	0.178	Mo8	O60	2.369	0.263
			5.437				5.838				5.897				5.875
Mo9	O50	1.694	1.820	Mo10	O53	1.684	1.871	Mo11	O24	1.680	1.893	Mo12	O64	1.675	1.920
Mo9	O21	1.773	1.452	Mo10	O6	1.809	1.309	Mo11	O9	1.980	0.801	Mo12	O63	1.836	1.212
Mo9	O26	1.886	1.051	Mo10	O70	1.814	1.289	Mo11	O10	1.982	0.797	Mo12	O62	1.861	1.130
Mo9	O49	1.969	0.828	Mo10	O8	2.043	0.670	Mo11	O1	2.023	0.710	Mo12	O13	2.037	0.682
Mo9	O4	2.108	0.555	Mo10	O17	2.083	0.598	Mo11	O3	2.027	0.701	Mo12	O43	2.071	0.619
Mo9	O2	2.511	0.175	Mo10	O46	2.369	0.263	Mo11	O2	2.085	0.593	Mo12	O78	2.437	0.217
									O21	2.214	0.410				
			5.881				5.999				5.906				5.778
Mo13	O57	1.682	1.885	Mo14	O80	1.680	1.893	Mo15	O71	1.676	1.917	Mo16	O85	1.673	1.932
Mo13	O17	1.995	0.769	Mo14	O66	1.842	1.191	Mo15	O66	1.903	1.001	Mo16	O55	1.911	0.978
Mo13	O8	2.007	0.742	Mo14	O68	1.852	1.159	Mo15	O62	1.937	0.908	Mo16	O27	1.952	0.870
Mo13	O47	2.009	0.737	Mo14	O41	2.028	0.699	Mo15	O51	1.994	0.771	Mo16	O51	1.982	0.797
Mo13	O13	2.024	0.707	Mo14	O69	2.048	0.659	Mo15	O79	2.015	0.726	Mo16	O18	2.000	0.758
Mo13	O43	2.067	0.625	Mo14	O86	2.377	0.257	Mo15	O78	2.271	0.348	Mo16	O60	2.269	0.351
	O22	2.208	0.417												
			5.883				5.858				5.671				5.686
Mo17	O84	1.675	1.925	Mo18	O67	1.702	1.779	Mo19	O58	1.668	1.962	Mo20	O82	1.680	1.896
Mo17	O63	1.912	0.974	Mo18	O31	1.857	1.143	Mo19	O28	2.000	0.758	Mo20	O52	1.804	1.328
Mo17	O68	1.952	0.870	Mo18	O312	1.857	1.142	Mo19	O412	2.008	0.740	Mo20	O522	1.804	1.328

Mo17	O61	1.964	0.840	Mo18	O52	2.099	0.570	Mo19	O41	2.008	0.740	Mo20	O692	2.050	0.657
Mo17	O52	2.024	0.707	Mo18	O5	2.099	0.570	Mo19	O69	2.034	0.687	Mo20	O69	2.050	0.656
Mo17	O86	2.299	0.322	Mo18	O11	2.219	0.405	Mo19	O692	2.034	0.687	Mo20	O16	2.749	0.089
								Mo19	O16	2.352	0.276				
			5.637				5.609				5.850				5.953
Mo21	O45	1.697	1.802	Mo22	O56	1.712	1.728	Mo23	O90	1.656	2.032	Mo24	O92	1.668	1.962
Mo21	O30	1.785	1.401	Mo22	O36	1.815	1.287	Mo23	O35	1.892	1.032	Mo24	O35	1.823	1.257
Mo21	O18	1.831	1.230	Mo22	O72	1.826	1.248	Mo23	O401	2.006	0.744	Mo24	O39	1.994	0.771
Mo21	O1	2.050	0.656	Mo22	O10	2.078	0.606	Mo23	O361	2.016	0.724	Mo24	O70	2.018	0.720
Mo21	O9	2.093	0.580	Mo22	O9	2.078	0.606	Mo23	O31	2.031	0.694	Mo24	O5	2.035	0.685
Mo21	O75	2.330	0.294	Mo22	O74	2.329	0.295	Mo23	O77	2.299	0.321	Mo24	O83	2.343	0.283
			5.962				5.770				5.547				5.679
Mo25	O87	1.693	1.826	Mo26	O25	1.683	1.878	Mo27	O94	1.695	1.817	Mo28	O88	1.701	1.783
Mo25	O932	1.920	0.953	Mo26	O39	1.777	1.433	Mo27	O932	1.935	0.913	Mo28	O65	1.793	1.372
Mo25	O72	1.976	0.811	Mo26	O42	1.825	1.252	Mo27	O93	1.935	0.913	Mo28	O79	1.854	1.151
Mo25	O44	1.979	0.803	Mo26	O8	2.068	0.623	Mo27	O65	2.004	0.748	Mo28	O28	2.057	0.644
Mo25	O30	2.033	0.689	Mo26	O47	2.099	0.571	Mo27	O652	2.004	0.748	Mo28	O41	2.078	0.606
Mo25	O89	2.239	0.382	Mo26	O12	2.350	0.277	Mo27	O91	2.245	0.376	Mo28	O76	2.276	0.343
			5.465				6.035				5.515				5.899
Mo29	O81	1.648	2.076	Mo30	O86	1.688	1.852								
Mo29	O20	1.812	1.298	Mo30	O76	1.759	1.512								
Mo29	O61	1.856	1.144	Mo30	O16	1.818	1.277								
Mo29	O17	2.062	0.634	Mo30	O95	1.991	0.778								
Mo29	O13	2.081	0.600												
Mo29	O95	2.646	0.119												
			5.872				5.419								

6. Thermogravimetric Analysis (TGA)

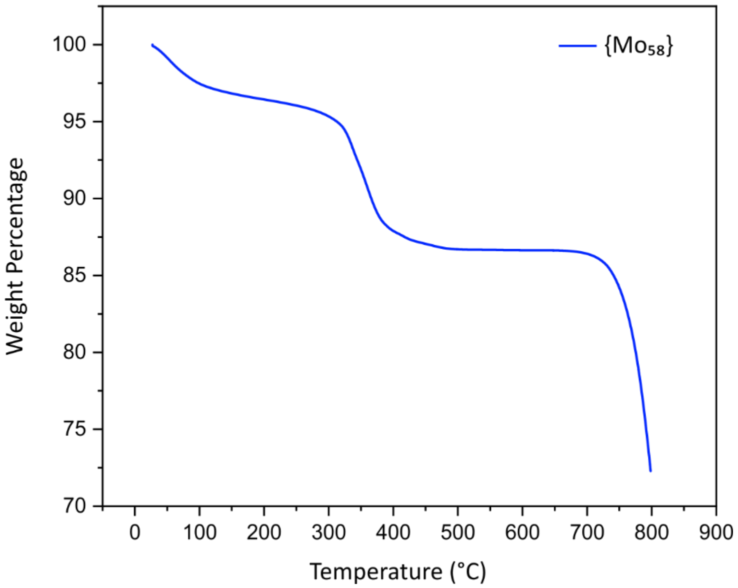

Figure S1. TGA Analysis of {Mo_{58}} **1** from RT to 800 ^{o}C. 6.3% weight loss from 0 – 200 ^{o}C corresponds to ~35 guest H_2O molecules. Number of H_2O molecules lost is approximately half that which is expected, this is due to the samples having been dried under vacuum.

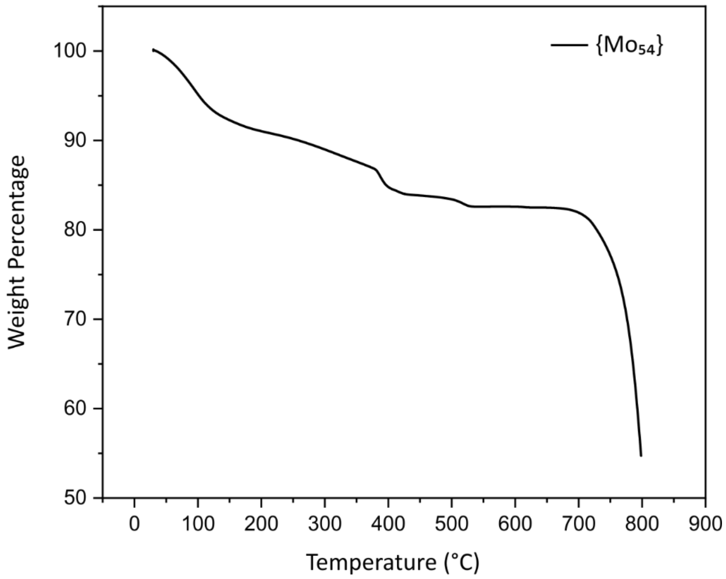

Figure S2. TGA Analysis of {Mo_{54}} **2** from RT to 800 ^{o}C. 9.5% weight loss from 0 – 200 ^{o}C corresponds to ~50 guest H_2O molecules.

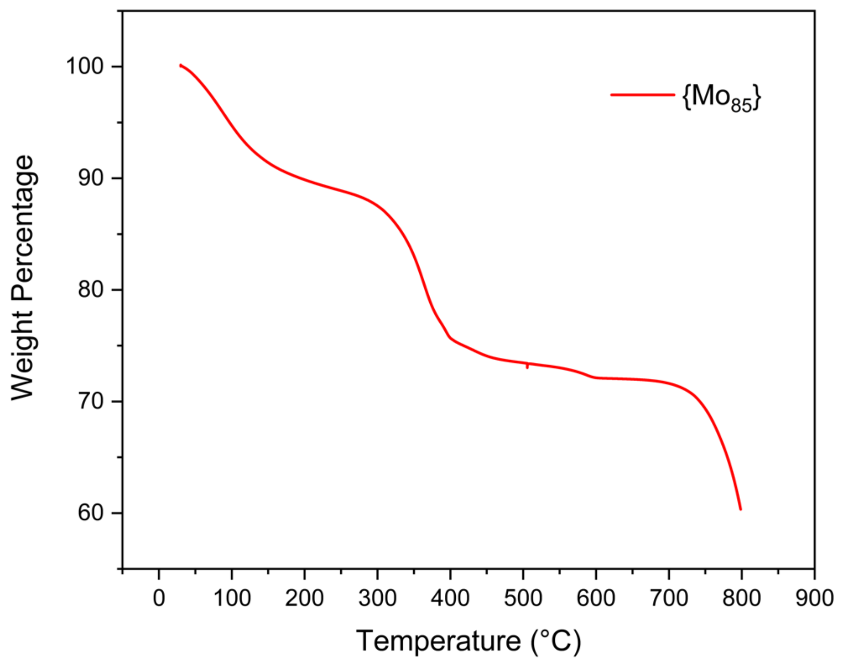

Figure S3. TGA Analysis of {Mo_{85}} **3** from RT to 800 ^{o}C. 10.6% weight loss from 0 – 200 ^{o}C which corresponds to ~85 guest H_2O molecules.

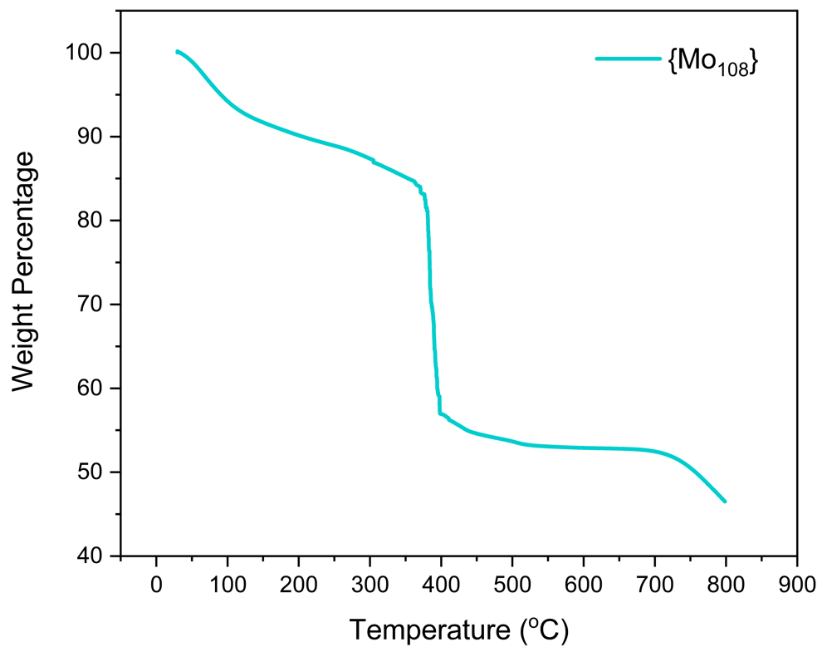

Figure S4. TGA Analysis of {Mo_{108}} **4** from RT to 800 ^{o}C. 10.0 % weight loss from 0 – 200 ^{o}C which corresponds to ~95 guest H_2O molecules.

7. EDX

Table S5. Experimentally derived elemental compositions of compound **1**.

{Mo_{58}}				
Spectrum Label	Spectrum 1 wt %	Spectrum 2 wt %	Spectrum 3 wt %	Average wt %
Mo	60.1	61.1	58.4	59.9
O	34.7	34.1	36.2	35.0
Na	0.40	0.30	0.30	0.33
S	4.80	4.50	5.10	4.80
N	0.00	0.00	0.00	0.00
Total	100.00	100.00	100.00	100.00

Table S6. Experimentally derived elemental compositions of compound **2**.

{Mo_{54}}				
Spectrum Label	Spectrum 1 wt %	Spectrum 2 wt %	Spectrum 3 wt %	Average wt %
Mo	61.4	63.4	65.7	63.5
O	32.0	32.7	29.0	31.2
Na	1.10	1.50	1.00	1.20
S	2.30	2.30	2.20	2.27
N	3.20	0.00	2.10	1.77
Total	100.00	100.00	100.00	100.00

Table S7. Experimentally derived elemental compositions of compound **3**

{Mo_{85}}				
Spectrum Label	Spectrum 1 wt %	Spectrum 2 wt %	Spectrum 3 wt %	Average wt %
Mo	71.2	68.8	70.1	70.0
O	24.7	27.0	25.5	25.7
Na	0.60	0.81	0.60	0.67
S	3.42	3.42	3.82	3.55
N	0.00	0.00	0.00	0.00
Total	100.00	100.00	100.00	100.00

Table S8. Experimentally derived elemental compositions of compound **4**

{Mo_{108}}				
Spectrum Label	Spectrum 1 wt %	Spectrum 2 wt %	Spectrum 3 wt %	Average wt %
Mo	64.2	63.5	66.7	64.8
O	34.5	34.4	31.6	33.5
Na	0	0.12	0	0.04
N	1.23	1.96	1.7	1.63
Total	100.00	100.00	100.00	100.00

Table S9. Theoretical and experimental values for elemental composition of compound **1**, with standard deviation of each element. Trace amounts of Na_2SO_4 believed to have co-crystallized with {Mo_{58}}. Mo and S emission spectrum peaks overlap, thus some deviations are expected for these two elements.

{Mo_{58}}			
Spectrum Label	Theoretical wt %	Experimental average wt %	Standard Deviation
Mo	59.2	59.9	0.7
O	36.8	35.0	1.8
Na	0.49	0.33	0.16
S	1.24	4.80	3.56
N	0.00	0.00	0.00

Table S10. Theoretical and experimental values for elemental composition of compound **2**, with standard deviation of each element. Trace amounts of Na_2SO_4 believed to have co-crystallized with {Mo_{54}}. Mo and S emission spectrum peaks overlap thus some deviations are expected for these two elements.

{Mo_{54}}			
Spectrum Label	Theoretical wt %	Experimental average wt %	Standard Deviation
Mo	61.5	63.5	2.00
O	34.8	31.2	3.60
Na	1.09	1.20	0.11
S	1.14	2.27	1.13
N	0.83	1.77	0.94

Table S11. Theoretical and experimental values for elemental composition of compound **3**, with standard deviation of each element. Mo and S emission spectrum peaks overlap, thus some deviations are expected for these two elements.

{Mo_{85}}			
Spectrum Label	Theoretical wt %	Experimental average wt %	Standard Deviation
Mo	63.3	70.0	6.3
O	34.3	25.7	8.6
Na	0.71	0.67	0.04
S	1.24	3.55	2.31
N	0.00	0.00	0.00

Table S12. Theoretical and experimental values for elemental composition of compound **4**, with standard deviation of each element. #

{Mo_{108}}			
Spectrum Label	Theoretical wt %	Experimental average wt %	Standard Deviation
Mo	63.1	64.8	1.7
O	34.9	33.5	1.4
Na	0.00	0.04	0.04
N	1.19	1.63	0.44

8. Structure Comparisons

Table S13. Comparison of compounds **1** – **4** with previously reported, representative, Mo blue species.

**Notation**	**Composition**	**Architecture**	**Reference**
{Mo_{54}}	$[Mo_{54}H_{33}O_{174}(SO_3)_3]^{9-}$	Wheel	This work
{Mo_{58}}	$[Mo_{58}H_{36}O_{180}(SO_4)_9]^{6-}$	Capped Wheel	This work
{Mo_{85}}	$[Mo_{85}H_{48}O_{267}S_2(SO_3)_3]^{16-}$	Capped Wheel	This work
{Mo_{90}}	$[Mo_{90}Ln_{10}O_{280}H_{10}(H_2O)_{80}]$	Wheel	9
{Mo_{102}}	$[Mo_{102}O_{282}(H_2O)_{78}(CH_3CO_2)_{12}]$	Ball	10
{Mo_{108}}	$[Mo_{108}H_{78}O_{358}]^{14-}$	Capped Wheel	This work
{Mo_{120}}	$[Mo_{120}Ln_6O_{366}H_{12}(H_2O)_{78}]^{6-}$	Wheel	11
{$Mo_{130}Ce_6$}	$[Mo_{130}Ce_6O_{396}H_{16.5}(H_2O)_{84}]^{1.5-}$	Wheel	12
C-{Mo_{132}}	$\{Mo_{132}O_{372}(OH)_{10}(H_2O)_{12}(SO_4)_5(CH_3COO)_{20}\}^{52-}$	Capped Wheel	13
L-{Mo_{132}}	$[Mo_{132}O_{382.4}H_{30}(H_2O)_{36}S_{9.6}(SO_4)_2]^{36-}$	Lantern	14
{Mo_{154}}	$[Mo_{154}O_{462}H_{14}(H_2O)_{70}]^{14-}$	Wheel	15
{Mo_{176}}	$[Mo_{176}O_{528}H_{16}(H_2O)_{80}]^{16-}$	Wheel	16
{$Mo_{180}(Orn)_{12}$}	$[Mo_{180}O_{536}H_{18}(H_2O)_{78}(C_5H_{13}N_2O_2)_7]^{6-}$	Capped Wheel	12
{Mo_{248}}	$[Mo_{248}O_{720}H_{16}(H_2O)_{128}]^{16-}$	Capped Wheel	17
{Mo_{368}}	$[Mo_{368}O_{1032}H_{16}(H_2O)_{240}(SO_4)_{48}]^{48-}$	Lemon	18

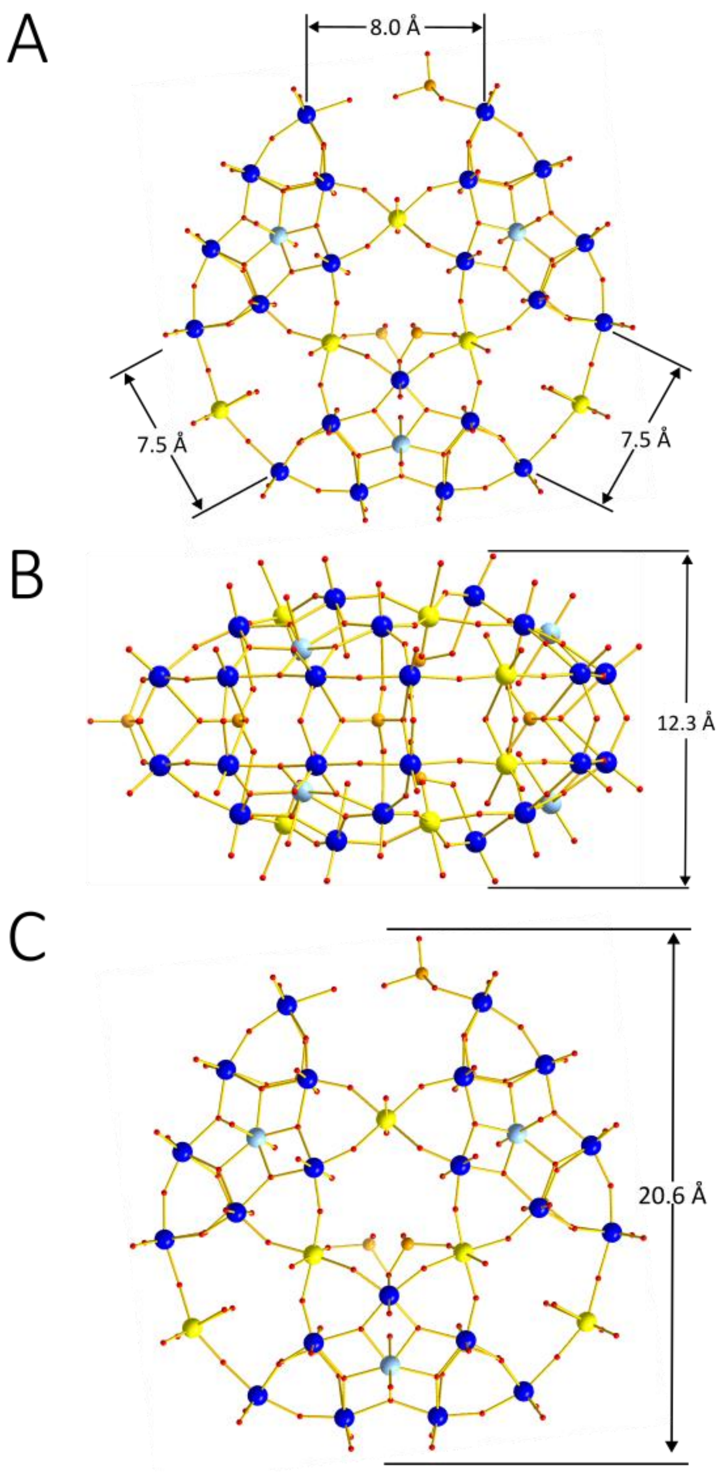

Figure S5. Ball and stick representation of {Mo_{58}} **1**. A) Upper and lower faces of {Mo_{58}} with the measured sizes of the filled and unfilled notches. B) Side view of {Mo_{58}} with cluster dimensions measured. C) Top view of {Mo_{58}} with cluster dimensions measured. Red atoms denote oxygen. Dark blue atoms denote Mo atoms contributing to {Mo_6}/ {Mo_8} units. Pale blue atoms denote Mo atoms occupying central positions of {Mo_6}/{Mo_8} units. Yellow atoms denote Mo atoms forming {Mo_1-b} or {Mo_2-e} bridging units. Orange atoms denote S.

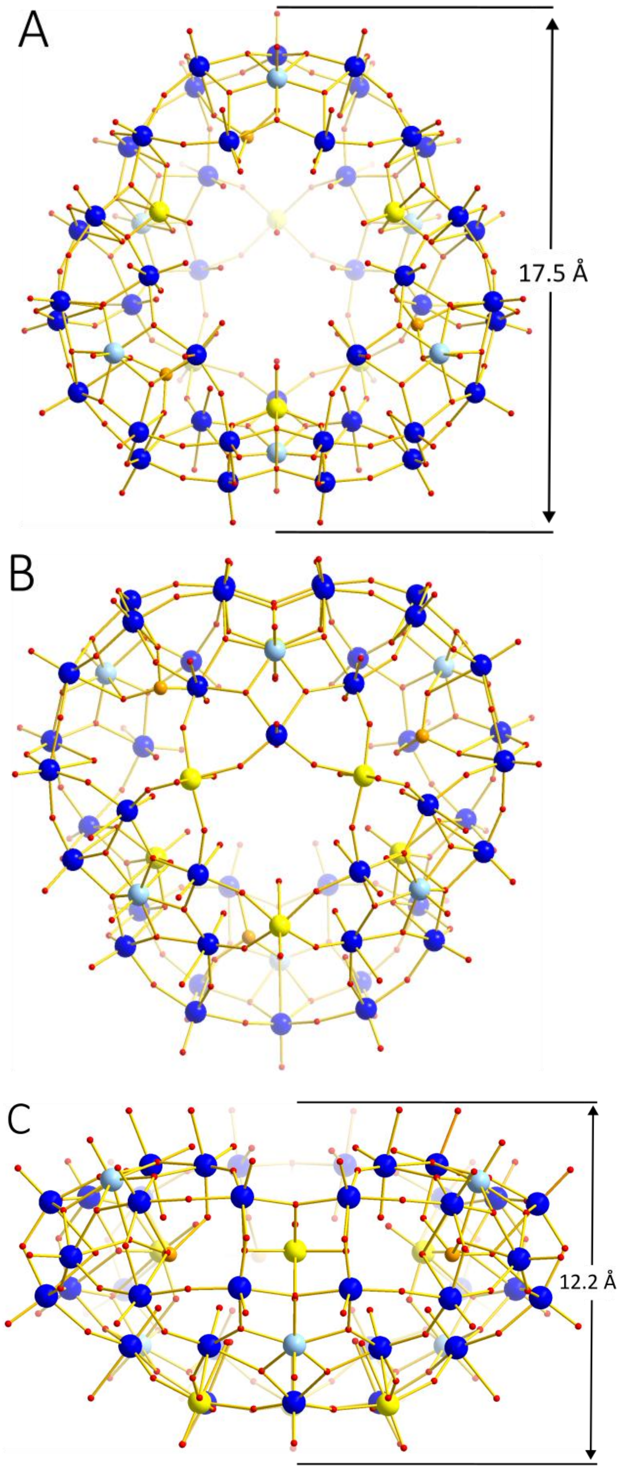

Figure S6. Ball and stick representation of $\{Mo_{54}\}$ **2** with cluster dimensions measured. A) Indicates top view of $\{Mo_{54}\}$. B) Indicates bottom view of $\{Mo_{54}\}$. C) Indicates side view of $\{Mo_{54}\}$. Red atoms denote oxygen. Dark blue atoms denote Mo atoms contributing to $\{Mo_6\}$/$\{Mo_8\}$ units. Pale blue atoms denote Mo atoms occupying central positions of $\{Mo_6\}$/$\{Mo_8\}$ units. Yellow atoms denote Mo atoms forming $\{Mo_1$-b$\}$ and $\{Mo_1$-s$\}$ units. Orange atoms denote S.

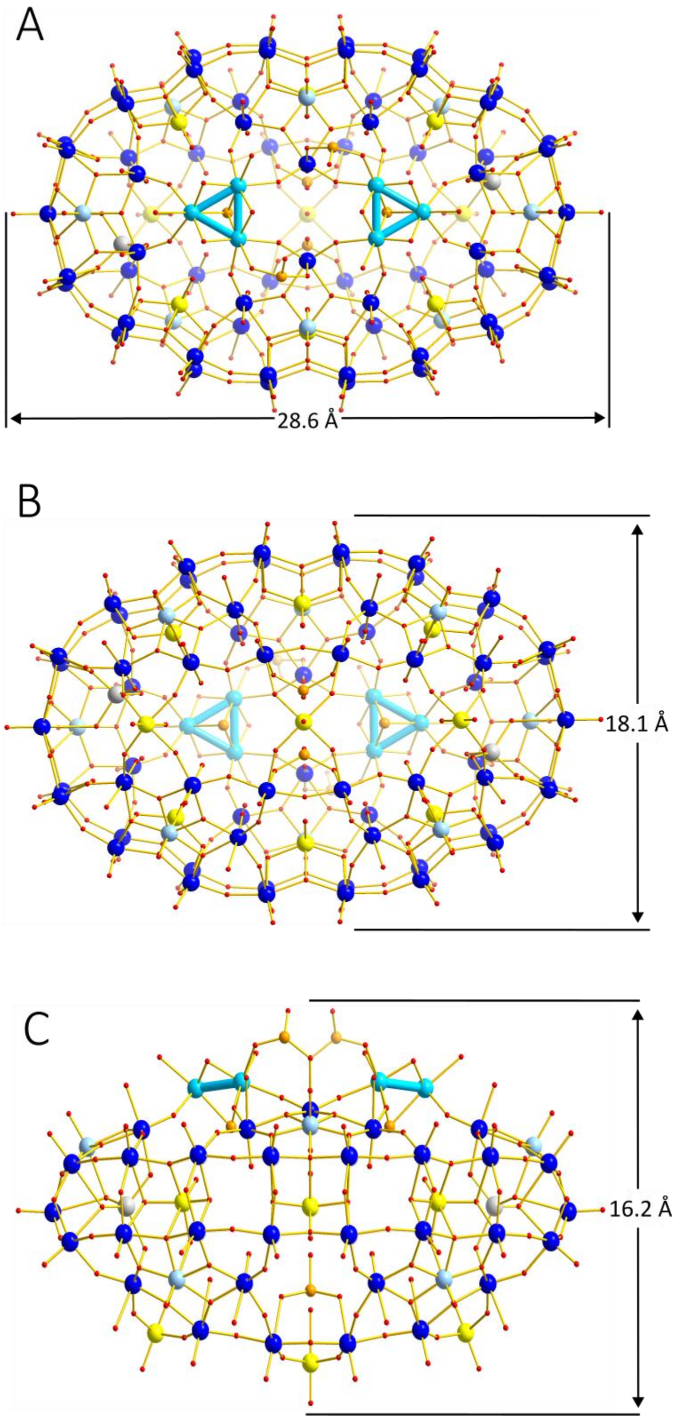

Figure S7. Ball and stick representation of {Mo_{85}} **3** with cluster dimensions measured. A) Top view of {Mo_{85}}. B) Bottom view. C) Side view. Red atoms denote oxygen. Dark blue atoms denote Mo atoms contributing to {Mo_6}/{Mo_8} units. Pale blue atoms denote Mo atoms occupying central positions of {Mo_6}/{Mo_8} units. Yellow atoms denote Mo atoms forming {Mo_1-b} and {Mo_1-s} units. Teal atoms denote Mo contributing to {Mo_3S} units. Gray atoms denote Mo contributing to {Mo_1-S} units behind bent pentagons. Orange atoms denote S.

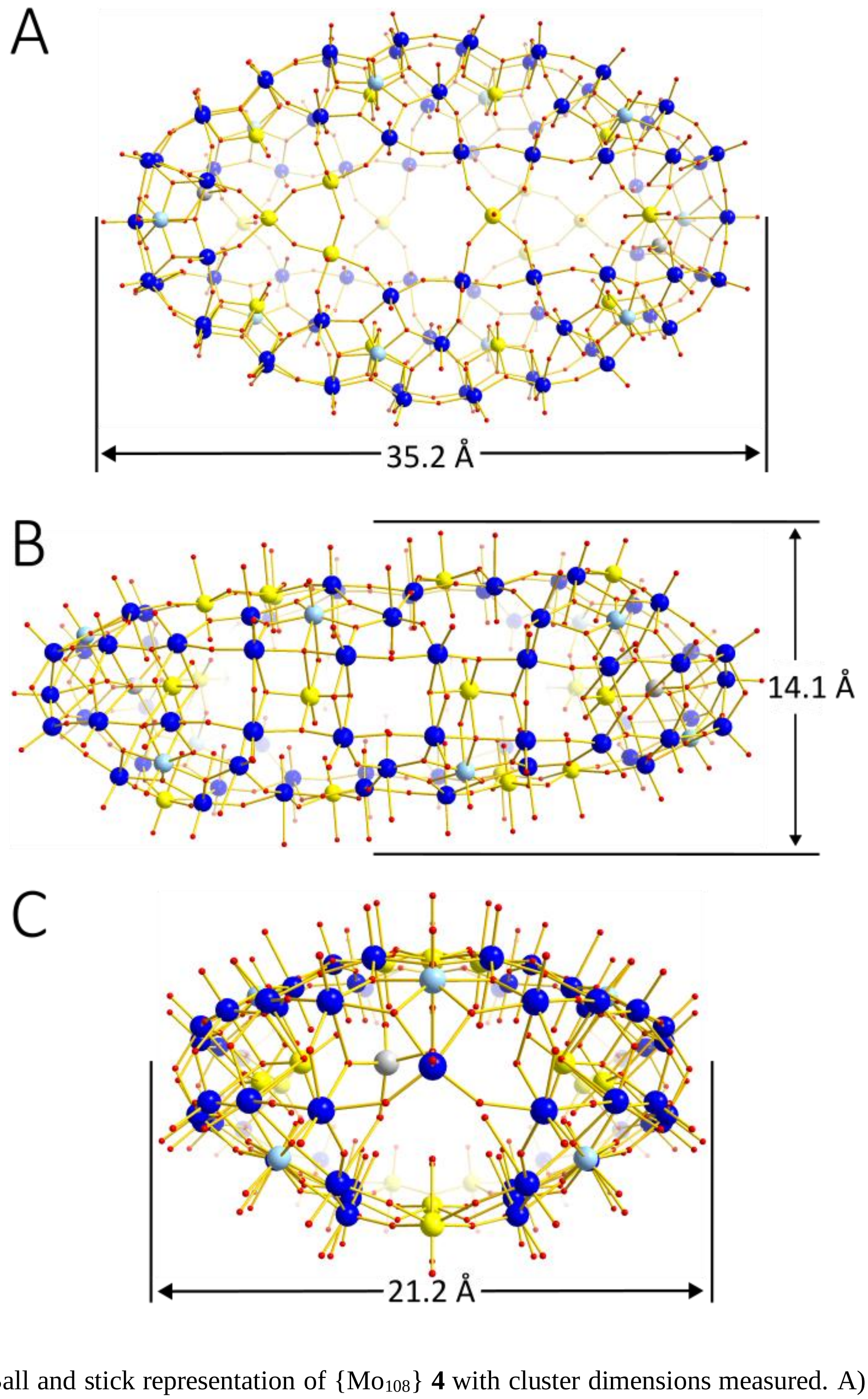

Figure S8. Ball and stick representation of {Mo_{108}} **4** with cluster dimensions measured. A) Top view of {Mo_{108}}. B) Side view. C) Front view. Red atoms denote oxygen. Dark blue atoms denote Mo atoms contributing to {Mo_6}/{Mo_8} units. Pale blue atoms denote Mo atoms occupying central positions of {Mo_6}/{Mo_8} units. Gray atoms denote Mo contributing to {Mo_1-S} units behind bent pentagons.

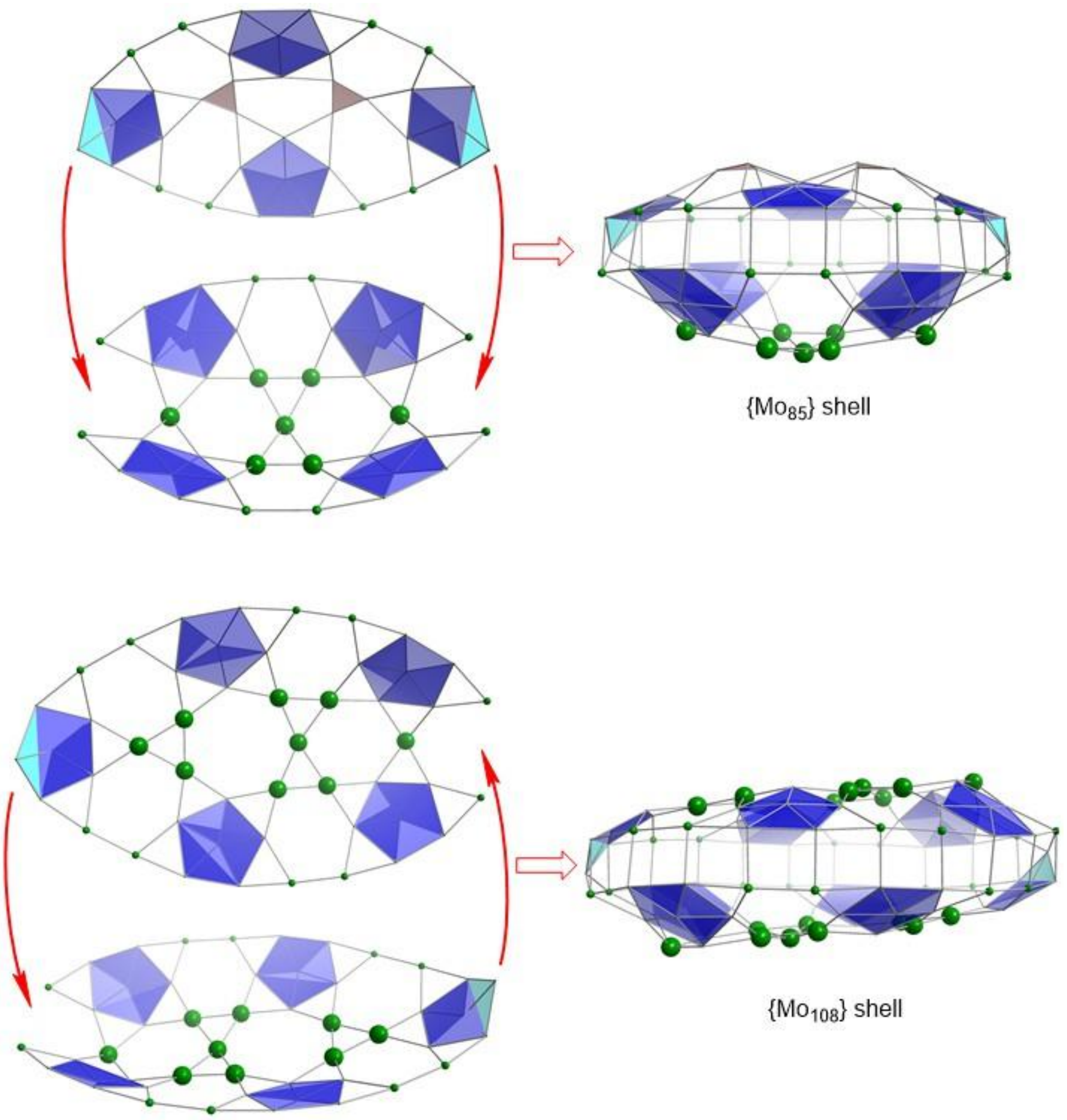

Figure S9. Mo-only framework presentations of the interlocking assemblies of $\{Mo_{85}\}$ and $\{Mo_{108}\}$ shells. Pentagon building blocks and bridging units are defined as in Figure 1 in paper. Brown triangles denote to the $\{Mo_3S\}$ bridging units. Mo atoms of the supporting units at backbones are omitted for clarity.

9. Electrochemical Measurement Results

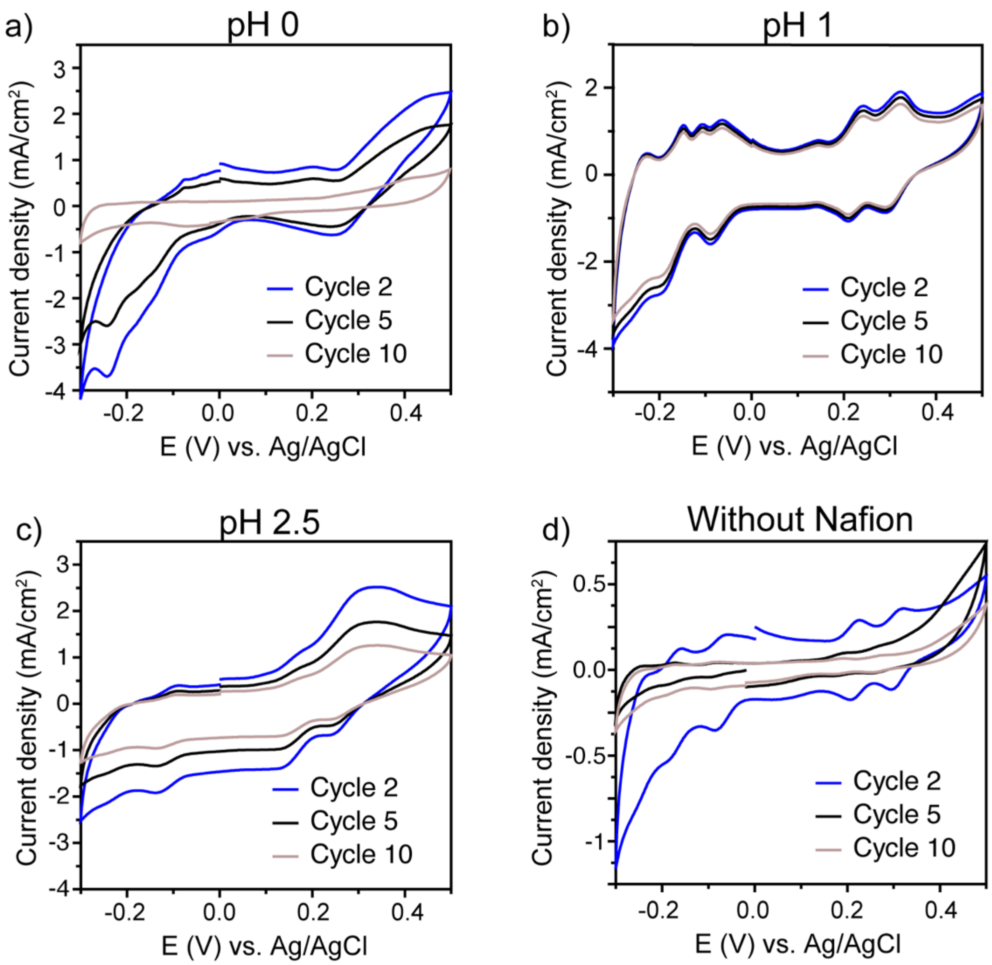

Figure S10. (a-c) CV measurements for the obtained {Mo_{54}} in N_2-saturated electrolyte at different pH. (d) CV measurements of the {Mo_{54}} electrode without adding Nafion solution in the experimental protocol. Scan rate of 50 mV s^{-1}. It is shown that at pH 1, the POM was stable enough to be measured by several cycles of CV. As well, the experimental protocol for fabrication of the electrode was carefully investigated to ensure the formation of a stable and uniform film on the GCE. The addition of carbon black serves to enhance electrical conductivity, while the Nafion film acts as a stabilizing binder, preventing the polyoxometalate (POM) material from dispersing into the electrolyte during operation. In these results, it can be seen that the electrode when measured without the Nafion, consistent results were not achieved as the POM might have been dispersed into the electrolyte.[19,20] The preparation method reflects our effort to balance material stability with optimal electrochemical performance.

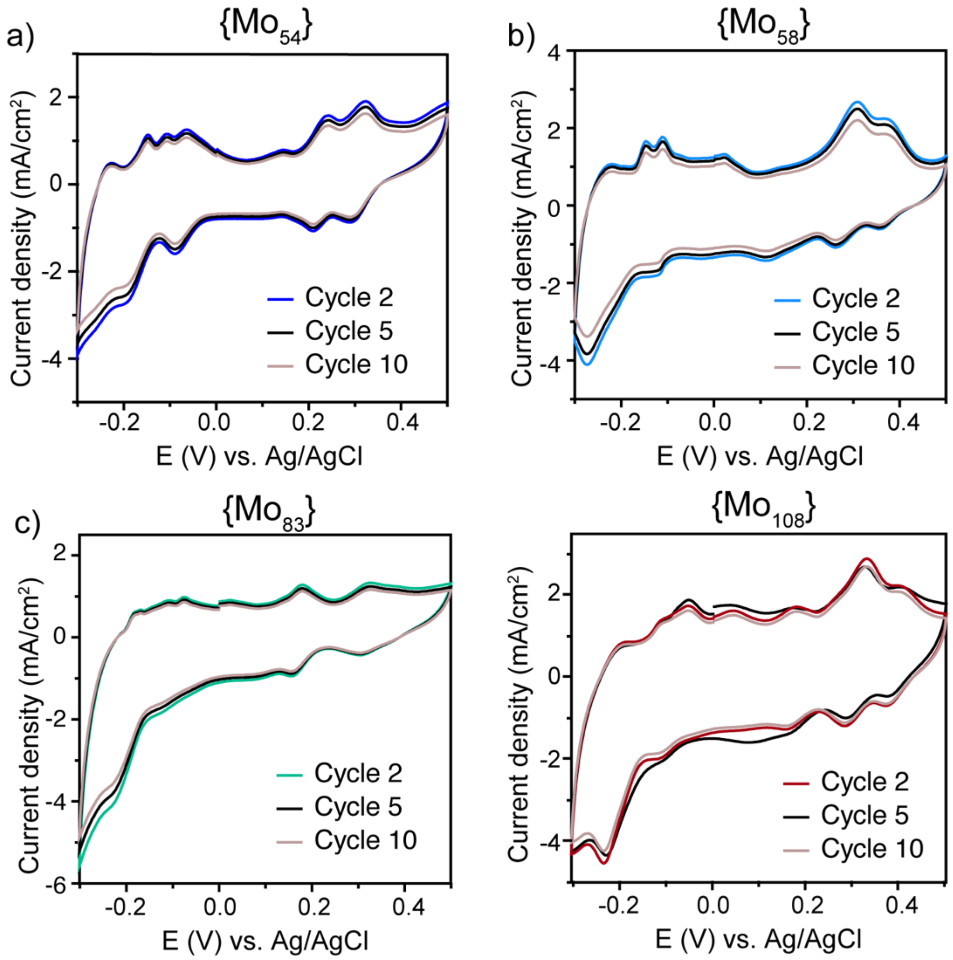

Figure S11. Cyclic voltammetry measurements for the obtained Mo-cluster in N2-saturated 0.05M H2SO4 (pH =1) electrolyte. Scan rate of 50 mV s-1. The electrodes at pH 1 shows CV measurements that consistently display well-resolved redox peaks. These peaks remain reproducible over multiple cycles, indicating that the POM structure is not degrading or undergoing significant structural changes during electrochemical cycling at pH 1.

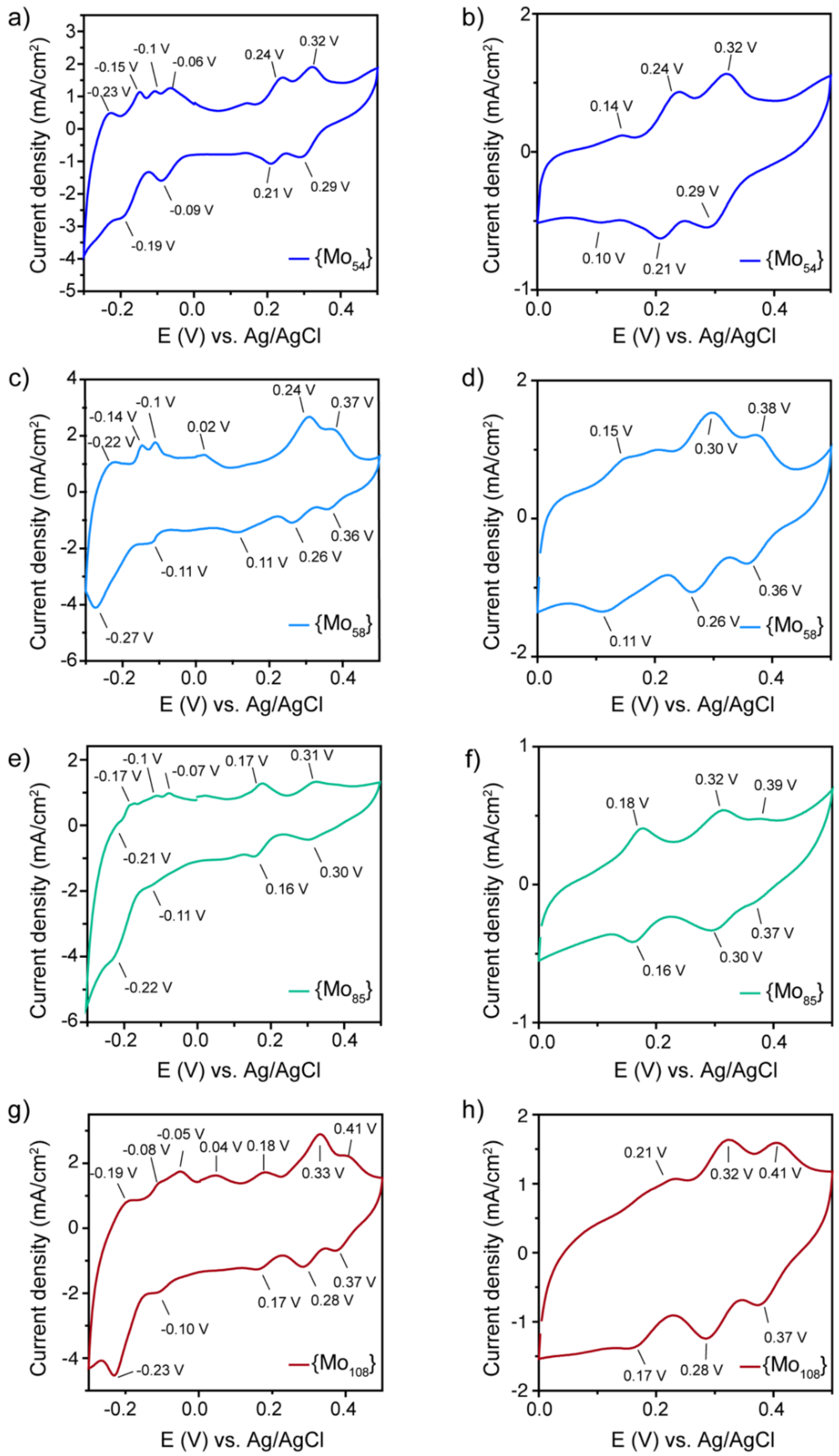

Figure S12. Cyclic voltammetry measurements for the obtained Mo-cluster in N_2-saturated 0.05M H_2SO_4 (pH =1) electrolyte. Scan rate of 50 mV s^{-1}.

Table S14. Electrochemical parameter values for the Mo-clusters in this work.

	**{Mo_{54}}**	**{Mo_{58}}**	**{Mo_{85}}**	**{Mo_{108}}**
**E_{pcMo1} (V)**	0.29	0.36	0.37	0.37
**E_{pcMo2} (V)**	0.21	0.26	0.30	0.28
**E_{pcMo3} (V)**	0.10	0.11	0.16	0.17
**E_{pcMo4} (V)**	-0.09	-0.11	-0.11	-0.10
**E_{pcMo5} (V)**	-0.19	-0.27	-0.22	-0.23
**ΔE_{pMo1} (V)**	0.03	0.02	0.02	0.04
**ΔE_{pMo2} (V)**	0.03	0.04	0.02	0.04
**ΔE_{pMo3} (V)**	0.04	0.04	0.02	0.04
**ΔE_{pMo4} (V)**	0.06	0.03	0.06	0.16
**ΔE_{pMo5} (V)**	0.05	0.05	0.02	0.04

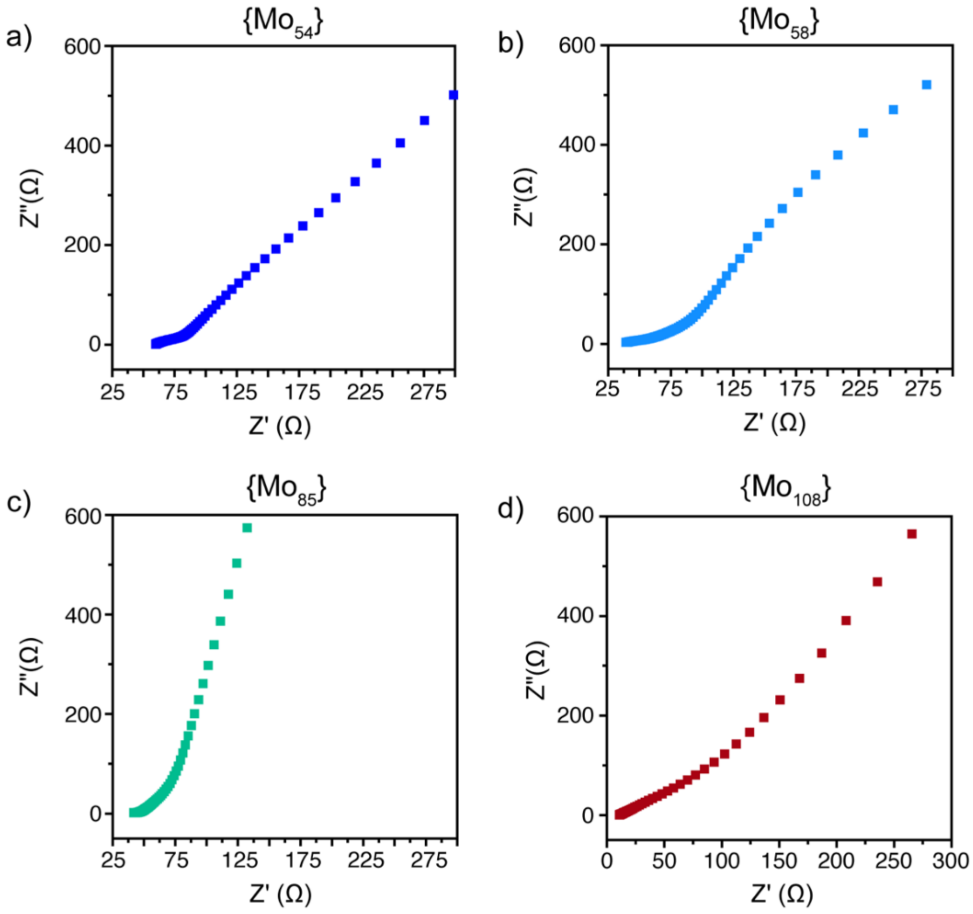

Figure S13. Electrochemical impedance spectroscopy (EIS) Nyquist plots of the different Mo-clusters.

Table S15. Z-fit equivalent circuit data for the Mo-clusters.

**Cluster**	**Rs (Ω)**	**Rr (Ω)**
$\{Mo_{54}\}$	60.1	22.3
$\{Mo_{58}\}$	39.3	38.2
$\{Mo_{85}\}$	41.8	11.0
$\{Mo_{108}\}$	10.9	9.5

References

(1) Sheldrick, G. M., SHELXT - Integrated space-group and crystal-structure determination. *Acta Crystallogr. Sect. A: Found. Crystallogr.* **2015**, *71*, 3-8.
(2) Sheldrick, G. M., Crystal structure refinement with SHELXL. *Acta Crystallogr. Sect. C: Cryst. Struct. Commun.* **2015**, *71*, 3-8.
(3) Dolomanov, O. V.; Bourhis, L. J.; Gildea, R. J.; Howard, J. A. K.; Puschmann, H., OLEX2: a complete structure solution, refinement and analysis program. *J. Appl. Crystallogr.* **2009**, *42*, 339-341.
(4) Farrugia, L. J., WinGX suite for small-molecule single-crystal crystallography. *J. Appl. Crystallogr.* **1999**, *32*, 837-838.
(5) Long, D. L.; Kögerler, P.; Cronin, L., Old clusters with new tricks:: Engineering S•••S interactions and novel physical properties in sulfite-based dawson clusters. *Angew. Chem. Int. Ed.* **2004**, *43*, 1817-1820.
(6) Spek, A. L., Single-crystal structure validation with the program PLATON. *J. Appl. Crystallogr.* **2002**, *36*, 7-13.
(7) Gagne, O. C.; Hawthorne, F. C., Comprehensive derivation of bond-valence parameters for ion pairs involving oxygen. *Acta Crystallogr. Sect. B: Struct. Sci.* **2015**, *71*, 562-578.
(8) Liu, W. T.; Thorp, H. H., Bond valence sum analysis of metal-ligand bond lengths in metalloenzymes and. 2. 2. Refined distances and other enzymes. *Inorg. Chem.* **1993**, *32*, 4102-4105.
(9)) Ribo, E. G.; Bell, N. L.; Xuan, W.; Luo, J.; Long, D.-L.; Liu, T.; Cronin, L., Synthesis, Assembly, and Sizing of Neutral, Lanthanide Substituted Molybdenum Blue Wheels $\{Mo_{90}Ln_{10}\}$. *J. Am. Chem. Soc.* **2020**, *142*, 17508-17514.
(10) Müller, A.; Shah, S. Q. N.; Bogge, H.; Schmidtmann, M.; Kögerler, P.; Hauptfleisch, B.; Leiding, S.; Wittler, K., Thirty electrons "trapped" in a spherical matrix: A molybdenum oxide-based nanostructured Keplerate reduced by 36 electrons. *Angew. Chem. Int. Ed.* **2000**, *39*, 1614-1616.
(11) Duros, V.; Grizou, J.; Xuan, W.; Hosni, Z.; Long, D.-L.; Miras, H. N.; Cronin, L., Human versus Robots in the Discovery and Crystallization of Gigantic Polyoxometalates. *Angew. Chem. Int. Ed.* **2017**, *56*, 10815-10820.
(12) Xuan, W.; Pow, R.; Long, D.-L.; Cronin, L., Exploring the Molecular Growth of Two Gigantic Half-Closed Polyoxometalate Clusters $\{Mo_{180}\}$ and $\{Mo_{130}Ce_6\}$. *Angew. Chem. Int. Ed.* **2017**, *56*, 9727-9731.
(13)) Li, X.-X.; Li, C.-H.; Hou, M.-J.; Zhu, B.; Chen, W.-C.; Sun, C.-Y.; Yuan, Y.; Guan, W.; Qin, C.; Shao, K.-Z.; Wang, X.-L.; Su, Z.-M., Ce-mediated molecular tailoring on gigantic polyoxometalate $\{Mo_{132}\}$ into half-closed $\{Ce_{11}Mo_{96}\}$ for high proton conduction. *Nature Commun.* **2023**, *14*, 5025.
(14) Liu, J.; Jiang, N.; Lin, J.-M.; Mei, Z.-B.; Dong, L.-Z.; Kuang, Y.; Liu, J.-J.; Yao, S.-J.; Li, S.-L.; Lan, Y.-Q., Structural Evolution of Giant Polyoxometalate: From "Keplerate" to "Lantern" Type Mo132 for Improved Oxidation Catalysis. *Angew. Chem. Int. Ed.* **2023**, *62*, e202304728.
(15) Müller, A.; Meyer, J.; Krickemeyer, E.; Diemann, E., Molybdenum blue: A 200 year old mystery unveiled. *Angew. Chem. Int. Ed. Engl.* **1996**, *35*, 1206-1208.
(16) Müller, A.; Krickemeyer, E.; Bogge, H.; Schmidtmann, M.; Beugholt, C.; Kögerler, P.; Lu, C. Z., Formation of a ring-shaped reduced "metal oxide" with the simple composition $(M0O_3)_{176}(H_2O)_{80}H_{32}$. *Angew. Chem. Int. Ed.* **1998**, *37*, 1220-1223.
(17) Muller, A.; Shah, S. Q. N.; Bogge, H.; Schmidtmann, M., Molecular growth from a Mo_{176} to a Mo_{248} cluster. *Nature* **1999**, *397*, 48-50.
(18) Müller, A.; Beckmann, E.; Bogge, H.; Schmidtmann, M.; Dress, A., Inorganic chemistry goes protein size: A Mo-368 nano-hedgehog initiating nanochemistry by symmetry breaking. *Angew. Chem. Int. Ed.* **2002**, *41*, 1162-1167.

(19) Fabre, B.; Falaise, C.; Cadot, E. Polyoxometalates-Functionalized Electrodes for (Photo)Electrocatalytic Applications: Recent Advances and Prospects. *ACS Catal*. **2022**, *12* (19), 12055–12091.
(20) Kim, Y.; Ketpang, K.; Jaritphun, S.; Park, J. S.; Shanmugam, S. A Polyoxometalate Coupled Graphene Oxide-Nafion Composite Membrane for Fuel Cells Operating at Low Relative Humidity †. *J. Mater. Chem. A*, **2015**, *3*, 8148-8155.
